# Supplementary material for: Primary Amine Nucleophilic Addition to Nitrilium Closo-Dodecaborate [B12H11NCCH3]−: A Simple and Effective Route to the New BNCT Drug Design
Source: Int J Mol Sci. 2021 Dec 13;22(24):13391. doi: 10.3390/ijms222413391 (PMC8707455; doi:10.3390/ijms222413391)

checkCIF/PLATON report

Structure factors have been supplied for datablock(s) Zh104

THIS REPORT IS FOR GUIDANCE ONLY. IF USED AS PART OF A REVIEW PROCEDURE FOR PUBLICATION, IT SHOULD NOT REPLACE THE EXPERTISE OF AN EXPERIENCED CRYSTALLOGRAPHIC REFEREE.

No syntax errors found. CIF dictionary Interpreting this report

Datablock: Zh104

Bond precision: C-C = 0.0021 A Wavelength=0.71073

Cell:	a=10.1707(2)	b=26.5124(6)	c=14.0828(3)
	alpha=90	beta=96.017(1)	gamma=90
Temperature:	100 K		

	Calculated	Reported
Volume	3776.50(14)	3776.50(14)
Space group	P 21/c	P 21/c
Hall group	-P 2ybc	-P 2ybc
Moiety formula	C24 H20 P, C5 H23 B12 N2, C3 H6 O3	?
Sum formula	C32 H49 B12 N2 O3 P	C32 H49 B12 N2 O3 P
Mr	670.42	670.42
Dx,g cm-3	1.179	1.179
Z	4	4
Mu (mm-1)	0.109	0.109
F000	1416.0	1416.0
F000’	1416.84	
h,k,lmax	14,37,19	14,37,19
Nref	11010	10981
Tmin,Tmax	0.962,0.978	
Tmin’	0.962	

Correction method= Not given

Data completeness= 0.997 Theta(max)= 29.998

R(reflections)= 0.0495(7561) wR2(reflections)= 0.1264(10981)

S = 1.026 Npar= 489

The following ALERTS were generated. Each ALERT has the format
test-name_ALERT_alert-type_alert-level.
Click on the hyperlinks for more details of the test.

Alert level B

PLAT910_ALERT_3_B Missing # of FCF Reflection(s) Below Theta(Min). 30 Note

Author Response: Closed by beamstop.

Alert level C

PLAT414_ALERT_2_C Short Intra D-H..H-X H2D ..H7 1.96 Ang.
x,y,z = 1_555 Check

Alert level G

PLAT007_ALERT_5_G Number of Unrefined Donor-H Atoms .... 2 Report
PLAT380_ALERT_4_G Incorrectly? Oriented X(sp2)-Methyl Moiety ..... C2 Check
PLAT978_ALERT_2_G Number C-C Bonds with Positive Residual Density. 12 Info

---

0 **ALERT level A** = Most likely a serious problem - resolve or explain
1 **ALERT level B** = A potentially serious problem, consider carefully
1 **ALERT level C** = Check. Ensure it is not caused by an omission or oversight
3 **ALERT level G** = General information/check it is not something unexpected

0 ALERT type 1 CIF construction/syntax error, inconsistent or missing data
2 ALERT type 2 Indicator that the structure model may be wrong or deficient
1 ALERT type 3 Indicator that the structure quality may be low
1 ALERT type 4 Improvement, methodology, query or suggestion
1 ALERT type 5 Informative message, check

It is advisable to attempt to resolve as many as possible of the alerts in all categories. Often the minor alerts point to easily fixed oversights, errors and omissions in your CIF or refinement strategy, so attention to these fine details can be worthwhile. In order to resolve some of the more serious problems it may be necessary to carry out additional measurements or structure refinements. However, the purpose of your study may justify the reported deviations and the more serious of these should normally be commented upon in the discussion or experimental section of a paper or in the "special_details" fields of the CIF. checkCIF was carefully designed to identify outliers and unusual parameters, but every test has its limitations and alerts that are not important in a particular case may appear. Conversely, the absence of alerts does not guarantee there are no aspects of the results needing attention. It is up to the individual to critically assess their own results and, if necessary, seek expert advice.

Publication of your CIF in IUCr journals

A basic structural check has been run on your CIF. These basic checks will be run on all CIFs submitted for publication in IUCr journals (*Acta Crystallographica*, *Journal of Applied Crystallography*, *Journal of Synchrotron Radiation*); however, if you intend to submit to *Acta Crystallographica Section C* or *E* or *IUCrData*, you should make sure that full publication checks are run on the final version of your CIF prior to submission.

Publication of your CIF in other journals

Please refer to the *Notes for Authors* of the relevant journal for any special instructions relating to CIF submission.

PLATON version of 06/01/2019; check.def file version of 19/12/2018

Datablock Zh104 - ellipsoid plot

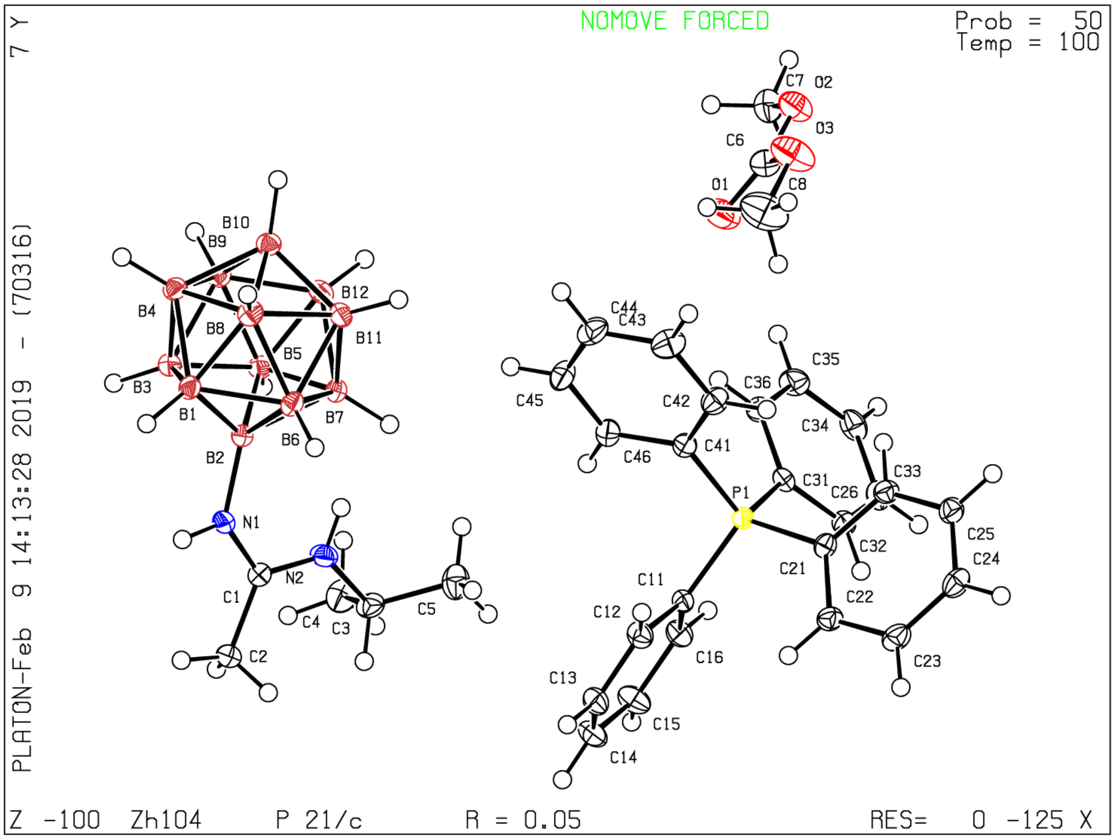

Supplement: Supplementary file 1 [file ijms-22-13391-s001.zip › checkcif_CCDC 2122677.pdf]
