# Supplementary material for: Primary Amine Nucleophilic Addition to Nitrilium Closo-Dodecaborate [B12H11NCCH3]−: A Simple and Effective Route to the New BNCT Drug Design
Source: Int J Mol Sci. 2021 Dec 13;22(24):13391. doi: 10.3390/ijms222413391 (PMC8707455; doi:10.3390/ijms222413391)

checkCIF/PLATON report

Structure factors have been supplied for datablock(s) zh108

THIS REPORT IS FOR GUIDANCE ONLY. IF USED AS PART OF A REVIEW PROCEDURE FOR PUBLICATION, IT SHOULD NOT REPLACE THE EXPERTISE OF AN EXPERIENCED CRYSTALLOGRAPHIC REFEREE.

No syntax errors found. CIF dictionary Interpreting this report

Datablock: zh108

```
Bond precision:  C-C = 0.0043 A          Wavelength=0.71073

Cell:            a=16.137(4)    b=14.573(3)        c=16.905(4)
                 alpha=90       beta=116.783(4)    gamma=90
Temperature:     100 K

                Calculated                 Reported
Volume          3549.0(14)                 3549.0(14)
Space group     P 21/c                     P 21/c
Hall group      -P 2ybc                    -P 2ybc
Moiety formula  C24 H20 P, C8 H27 B12 N2   ?
Sum formula     C32 H47 B12 N2 P           C32 H47 B12 N2 P
Mr              620.41                     620.40
Dx,g cm-3       1.161                      1.161
Z               4                          4
Mu (mm-1)       0.104                      0.104
F000            1312.0                     1312.0
F000'           1312.72
h,k,lmax        20,18,21                   20,18,21
Nref            8143                       8076
Tmin,Tmax       0.979,0.998
Tmin'           0.979

Correction method= Not given

Data completeness= 0.992          Theta(max)= 27.499

R(reflections)= 0.0633( 3875)     wR2(reflections)= 0.1418( 8076)

S = 0.939                  Npar= 458
```

The following ALERTS were generated. Each ALERT has the format
test-name_ALERT_alert-type_alert-level.
Click on the hyperlinks for more details of the test.

Alert level C

```
RINTA01_ALERT_3_C  The value of Rint is greater than 0.12
            Rint given   0.136
PLAT026_ALERT_3_C Ratio Observed / Unique Reflections (too) Low ..        48% Check
PLAT340_ALERT_3_C Low Bond Precision on  C-C Bonds ...............    0.00429 Ang.
PLAT411_ALERT_2_C Short Inter H...H Contact  H5       ..H43A     .       2.10 Ang.
                                                 1-x,1-y,-z  =      3_665 Check
PLAT414_ALERT_2_C Short Intra D-H..H-X       H1       ..H2A             1.98 Ang.
                                                      x,y,z  =      1_555 Check
```

Alert level G

```
PLAT007_ALERT_5_G Number of Unrefined Donor-H Atoms ..............          2 Report
PLAT020_ALERT_3_G The Value of Rint is Greater Than 0.12 .........      0.136 Report
PLAT380_ALERT_4_G Incorrectly? Oriented X(sp2)-Methyl Moiety .....         C2 Check
PLAT883_ALERT_1_G No Info for _atom_sites_solution_primary .......     Please Do !
PLAT933_ALERT_2_G Number of OMIT Records in Embedded .res File ...          2 Note
```

---

0 **ALERT level A** = Most likely a serious problem - resolve or explain
0 **ALERT level B** = A potentially serious problem, consider carefully
5 **ALERT level C** = Check. Ensure it is not caused by an omission or oversight
5 **ALERT level G** = General information/check it is not something unexpected

1 ALERT type 1 CIF construction/syntax error, inconsistent or missing data
3 ALERT type 2 Indicator that the structure model may be wrong or deficient
4 ALERT type 3 Indicator that the structure quality may be low
1 ALERT type 4 Improvement, methodology, query or suggestion
1 ALERT type 5 Informative message, check

It is advisable to attempt to resolve as many as possible of the alerts in all categories. Often the minor alerts point to easily fixed oversights, errors and omissions in your CIF or refinement strategy, so attention to these fine details can be worthwhile. In order to resolve some of the more serious problems it may be necessary to carry out additional measurements or structure refinements. However, the purpose of your study may justify the reported deviations and the more serious of these should normally be commented upon in the discussion or experimental section of a paper or in the "special_details" fields of the CIF. checkCIF was carefully designed to identify outliers and unusual parameters, but every test has its limitations and alerts that are not important in a particular case may appear. Conversely, the absence of alerts does not guarantee there are no aspects of the results needing attention. It is up to the individual to critically assess their own results and, if necessary, seek expert advice.

Publication of your CIF in IUCr journals

A basic structural check has been run on your CIF. These basic checks will be run on all CIFs submitted for publication in IUCr journals (*Acta Crystallographica*, *Journal of Applied Crystallography*, *Journal of Synchrotron Radiation*); however, if you intend to submit to *Acta Crystallographica Section C* or *E* or *IUCrData*, you should make sure that full publication checks are run on the final version of your CIF prior to submission.

Publication of your CIF in other journals

Please refer to the *Notes for Authors* of the relevant journal for any special instructions relating to CIF submission.

PLATON version of 18/02/2019; check.def file version of 18/02/2019

Datablock zh108 - ellipsoid plot

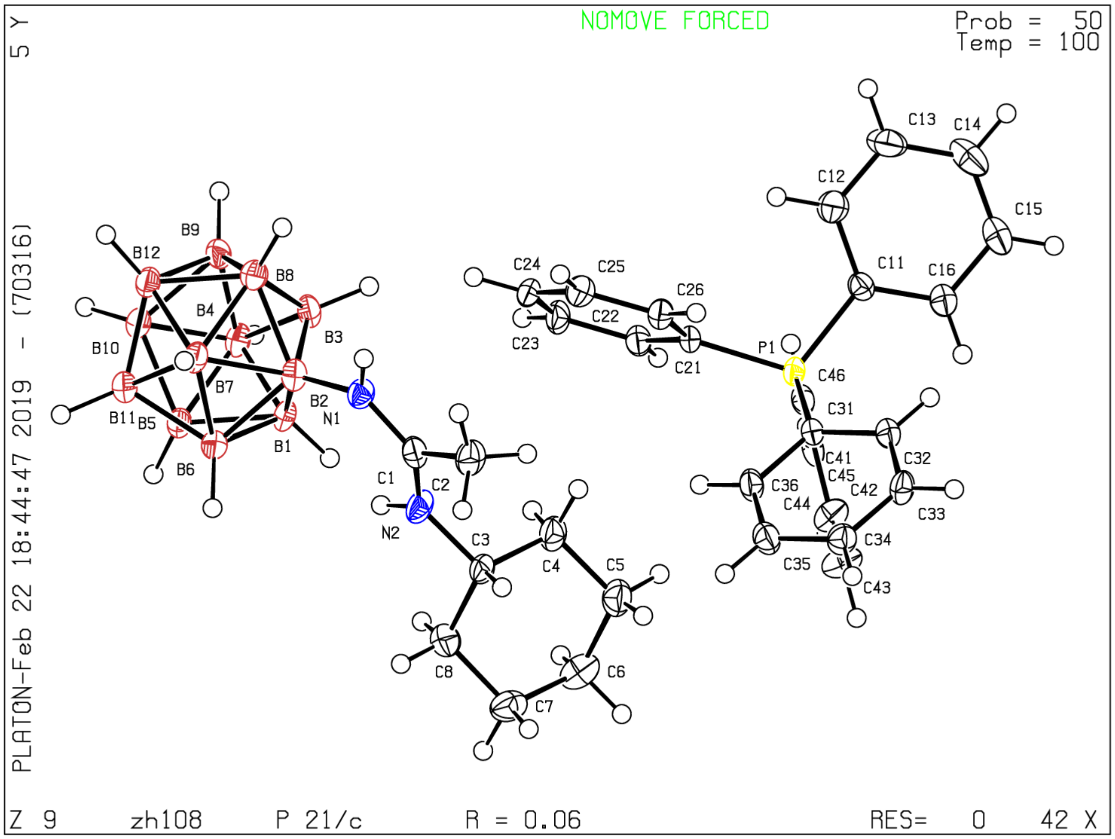

Supplement: Supplementary file 1 [file ijms-22-13391-s001.zip › checkcif_CCDC 2122678.pdf]
