# Supplementary material for: Primary Amine Nucleophilic Addition to Nitrilium Closo-Dodecaborate [B12H11NCCH3]−: A Simple and Effective Route to the New BNCT Drug Design
Source: Int J Mol Sci. 2021 Dec 13;22(24):13391. doi: 10.3390/ijms222413391 (PMC8707455; doi:10.3390/ijms222413391)
Supplement: Supplementary file 1 [file ijms-22-13391-s001.zip › nelyubin_supplementary_v2.pdf]

Supplementary Material

1. Experimental setup for preparation of $(NBu_4)[B_{12}H_{11}(NCCH_3)]$.

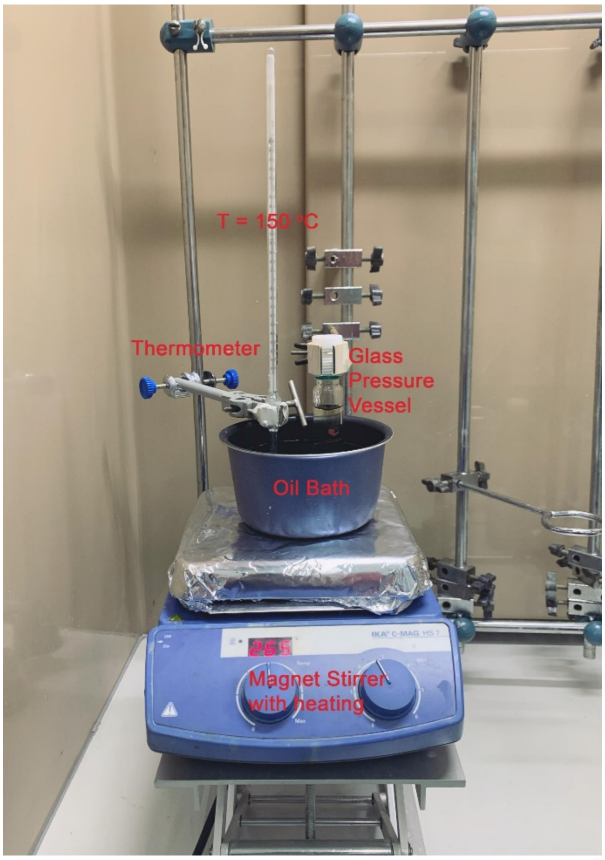

Reaction mixture after synthesis.

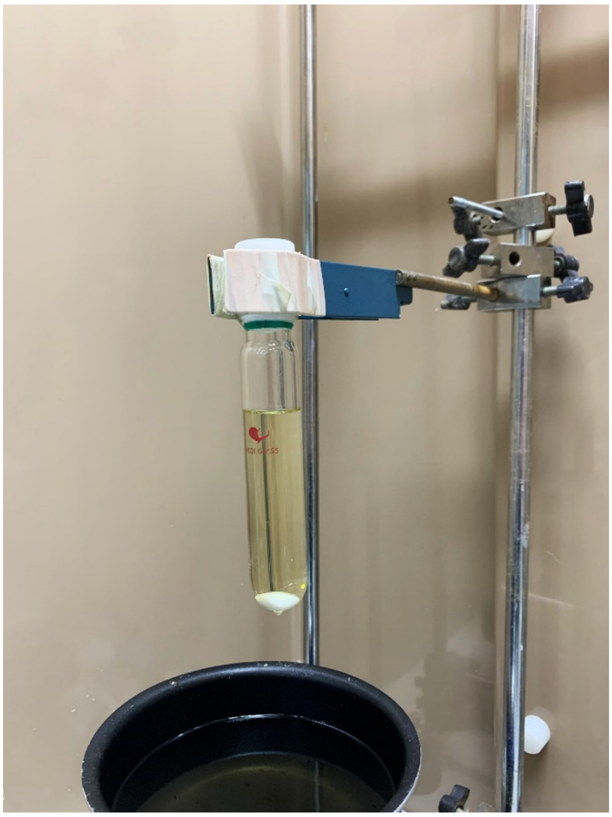

Spectral data for synthesised compounds.

^{11}B{^{1}H} NMR spectrum $(NBu_4)[B_{12}H_{11}(NCCH_3)]$ 2.

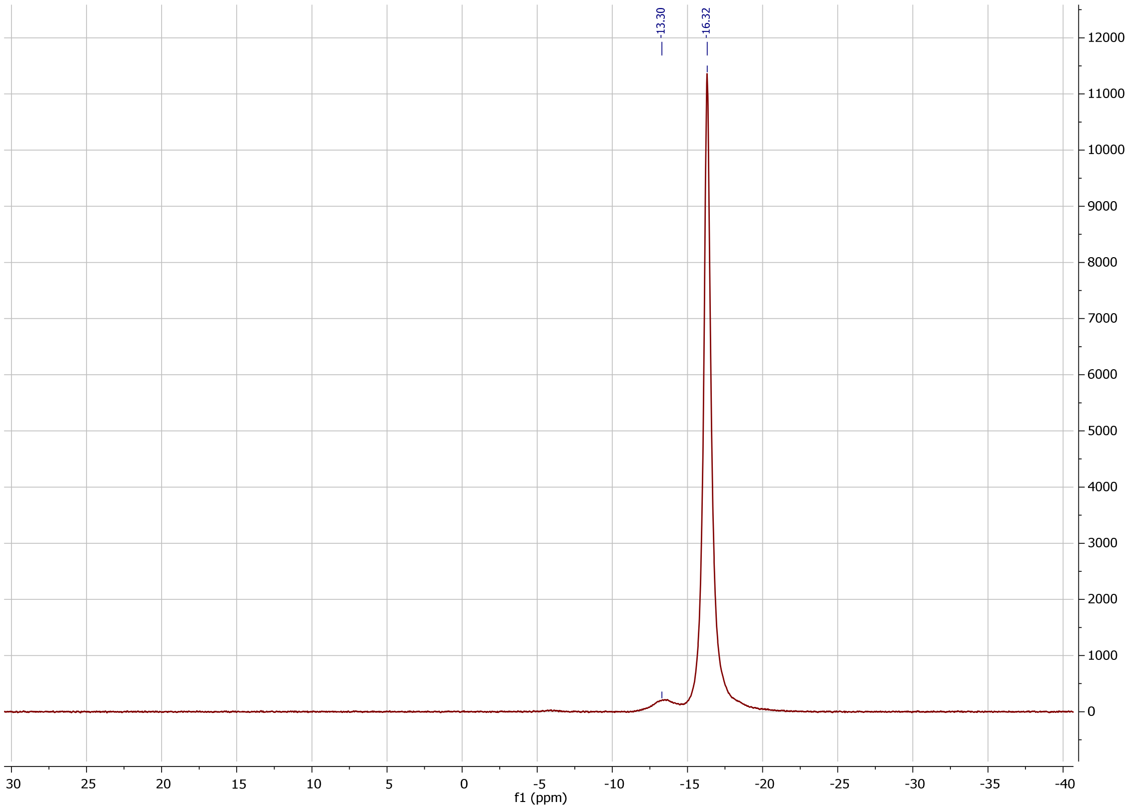

^{1}H NMR spectrum $(NBu_4)[B_{12}H_{11}(NCCH_3)]$ 2.

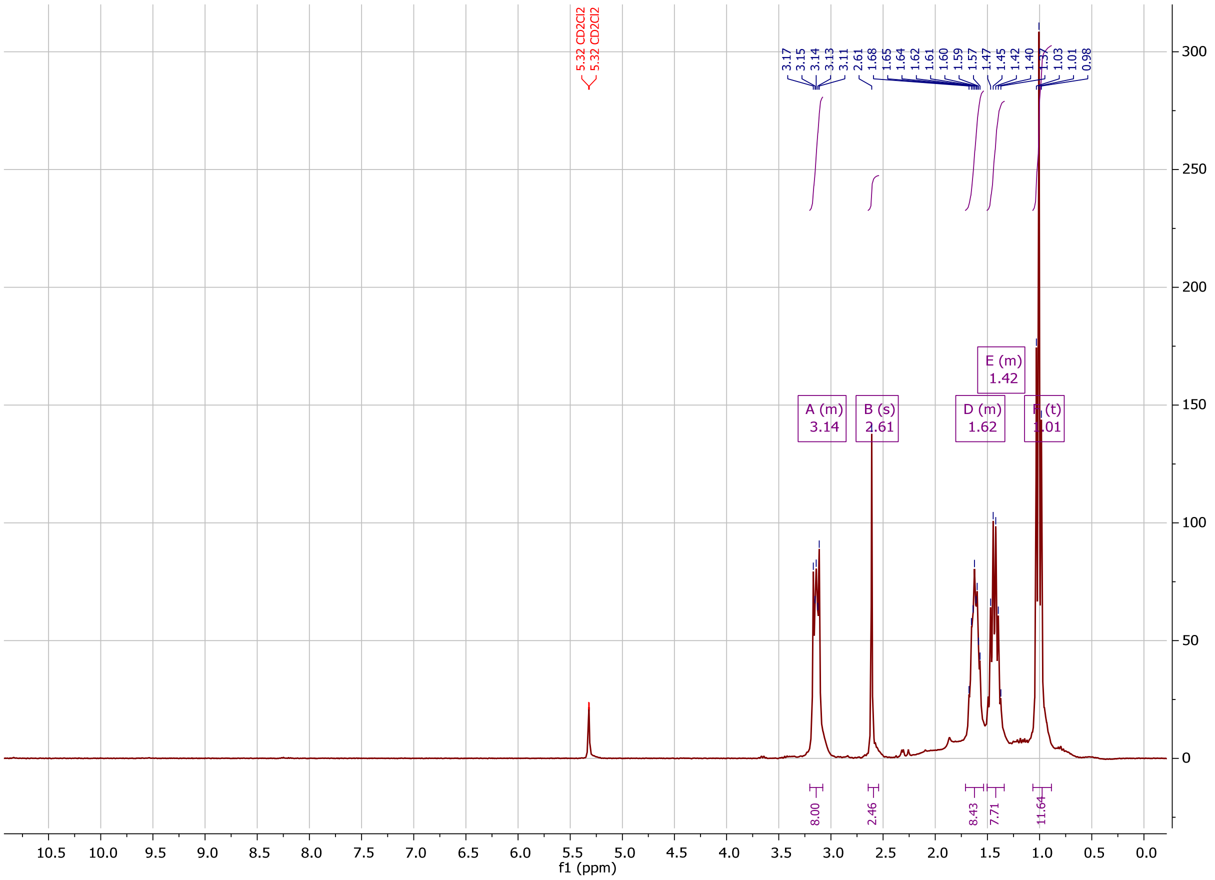

^{13}C NMR spectrum $(NBu_4)[B_{12}H_{11}(NCCH_3)]$ 2.

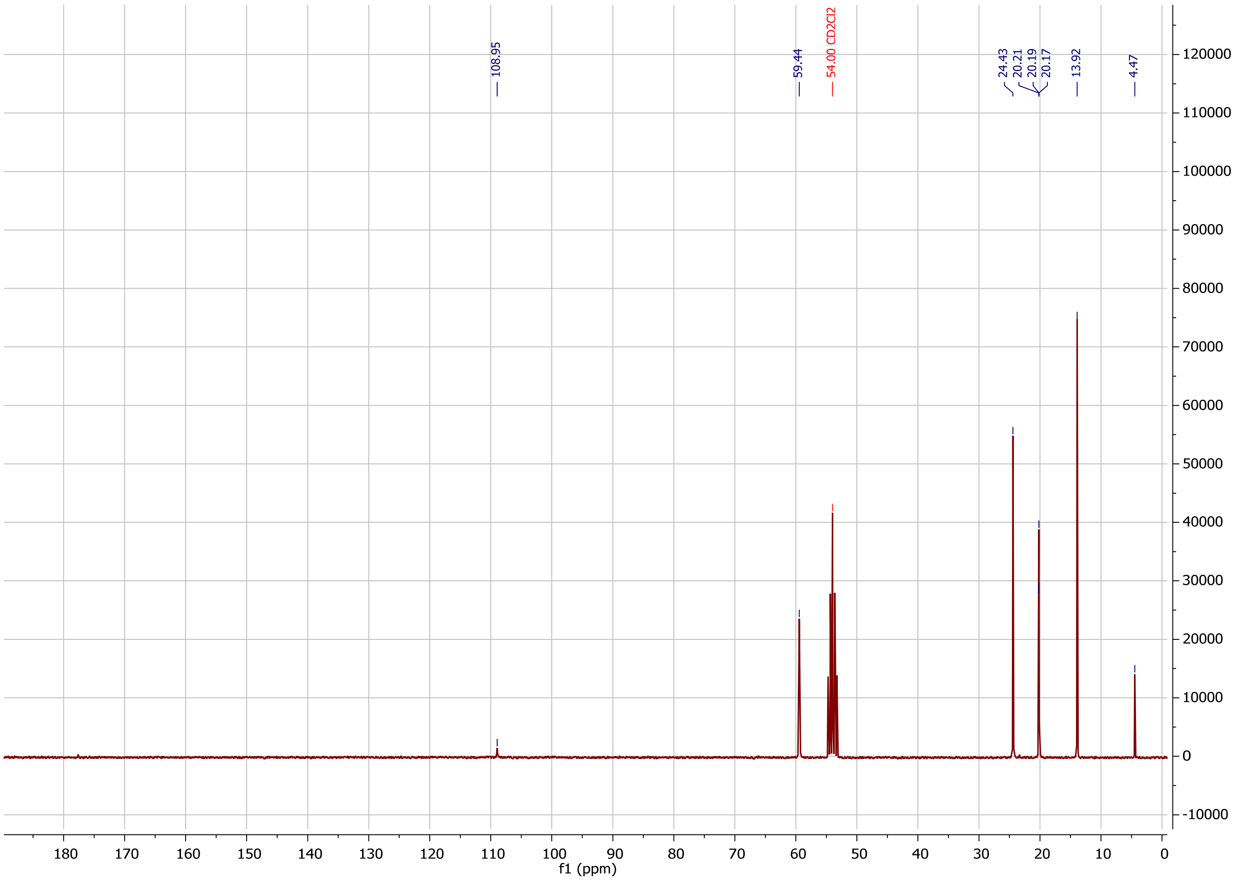

ESI-MS spectrum $(NBu_4)[B_{12}H_{11}(NCCH_3)]$ (negative area) 2.

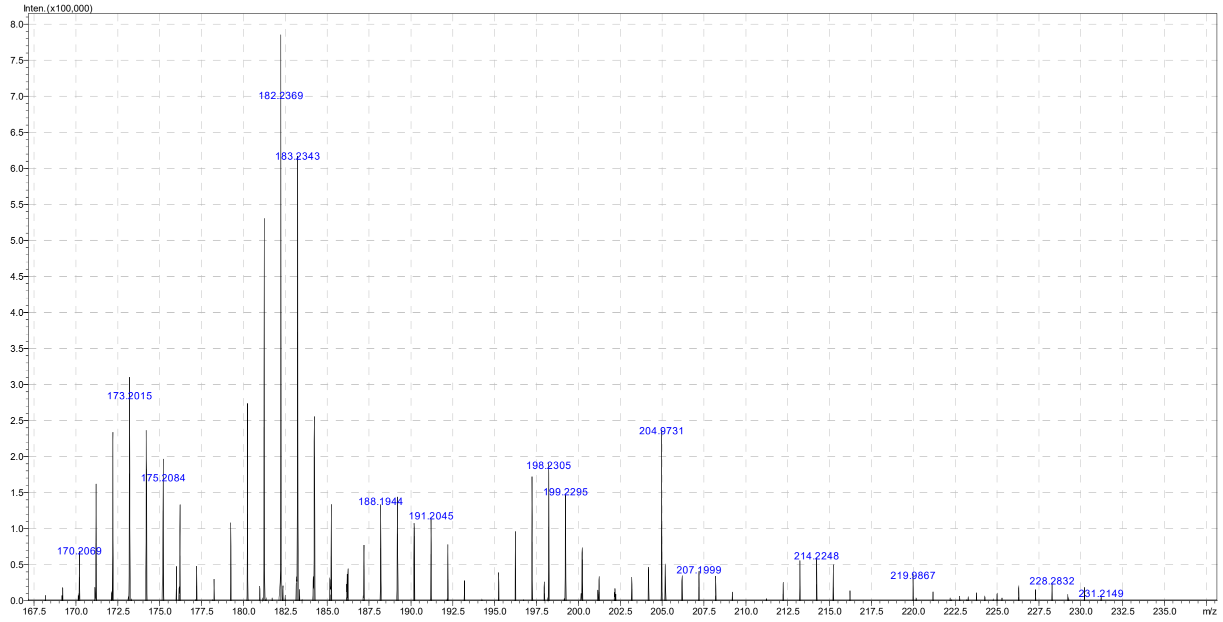

$^{11}B\{^{1}H\}$ NMR spectrum $(NBu_4)[B_{12}H_{11}(NHC(NH_2)CH_3)]$ 3a.

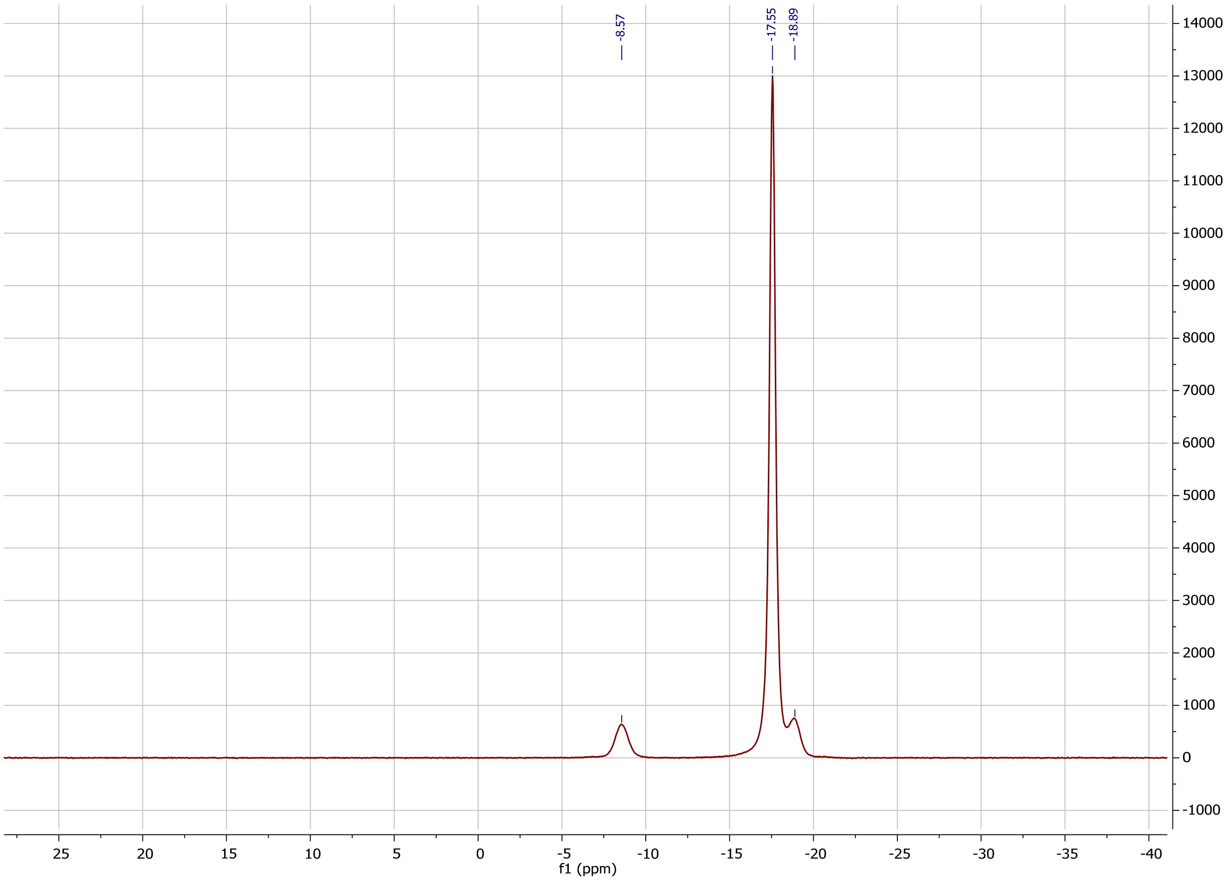

^{1}H NMR spectrum $(NBu_4)[B_{12}H_{11}(NHC(NH_2)CH_3)]$ 3a.

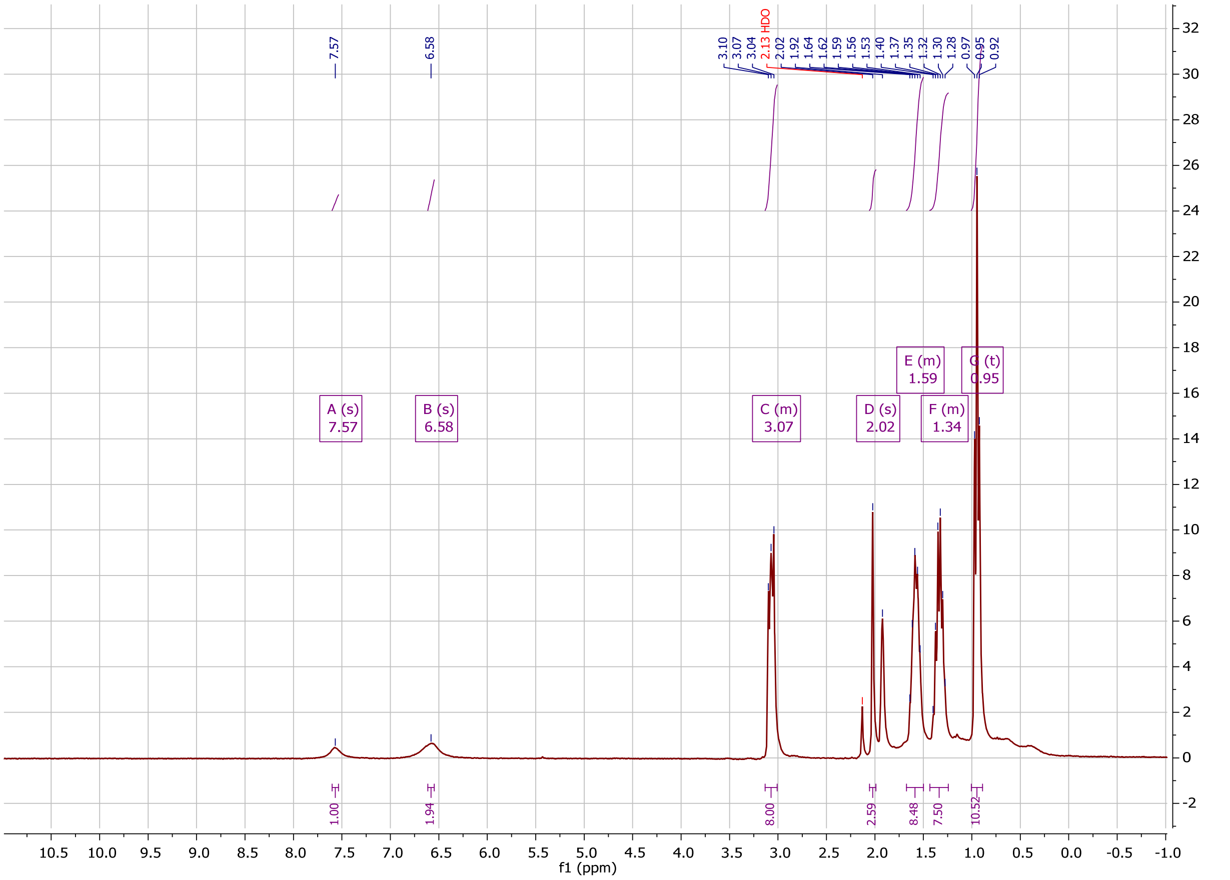

^{13}C NMR spectrum $(NBu_4)[B_{12}H_{11}(NHC(NH_2)CH_3)]$ 3a.

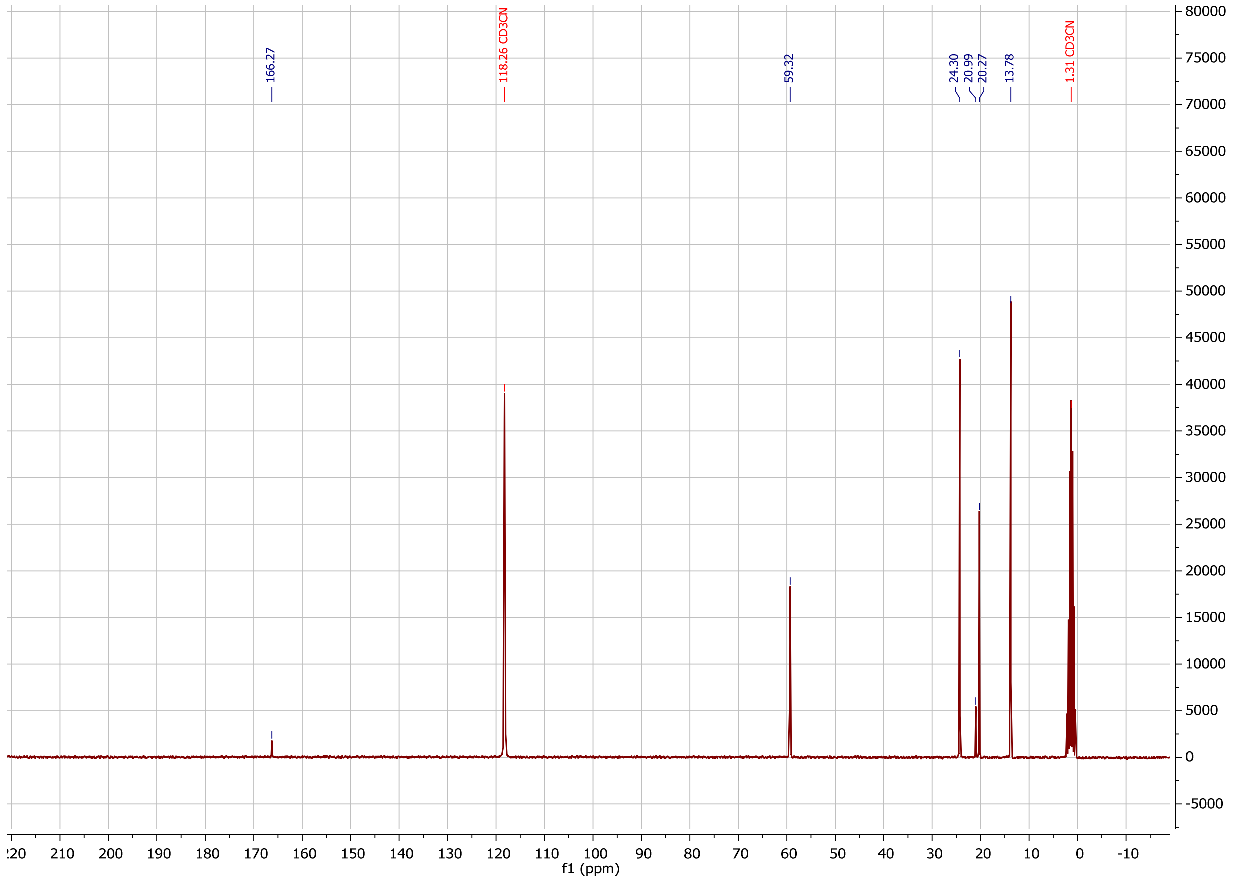

ESI-MS spectrum $(NBu_4)[B_{12}H_{11}(NHC(NH_2)CH_3)]$ (negative area) 3a.

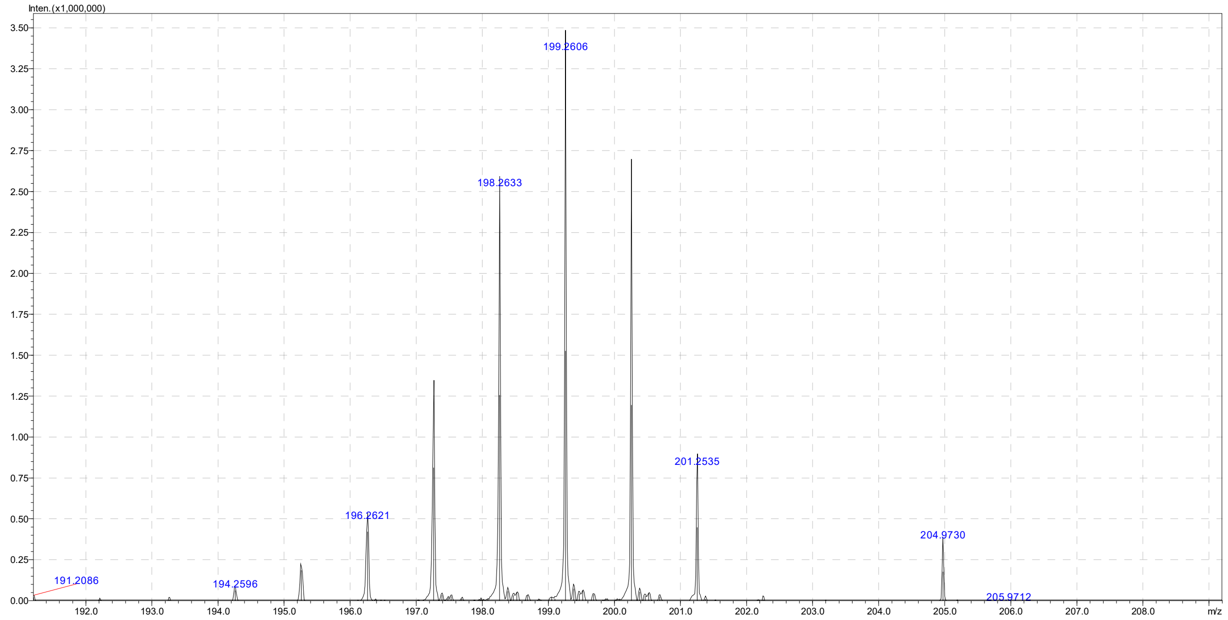

$^{11}B\{^{1}H\}$ NMR spectrum $(NBu_4)[B_{12}H_{11}(NHC(NHCH_3)CH_3)]$ 3b.

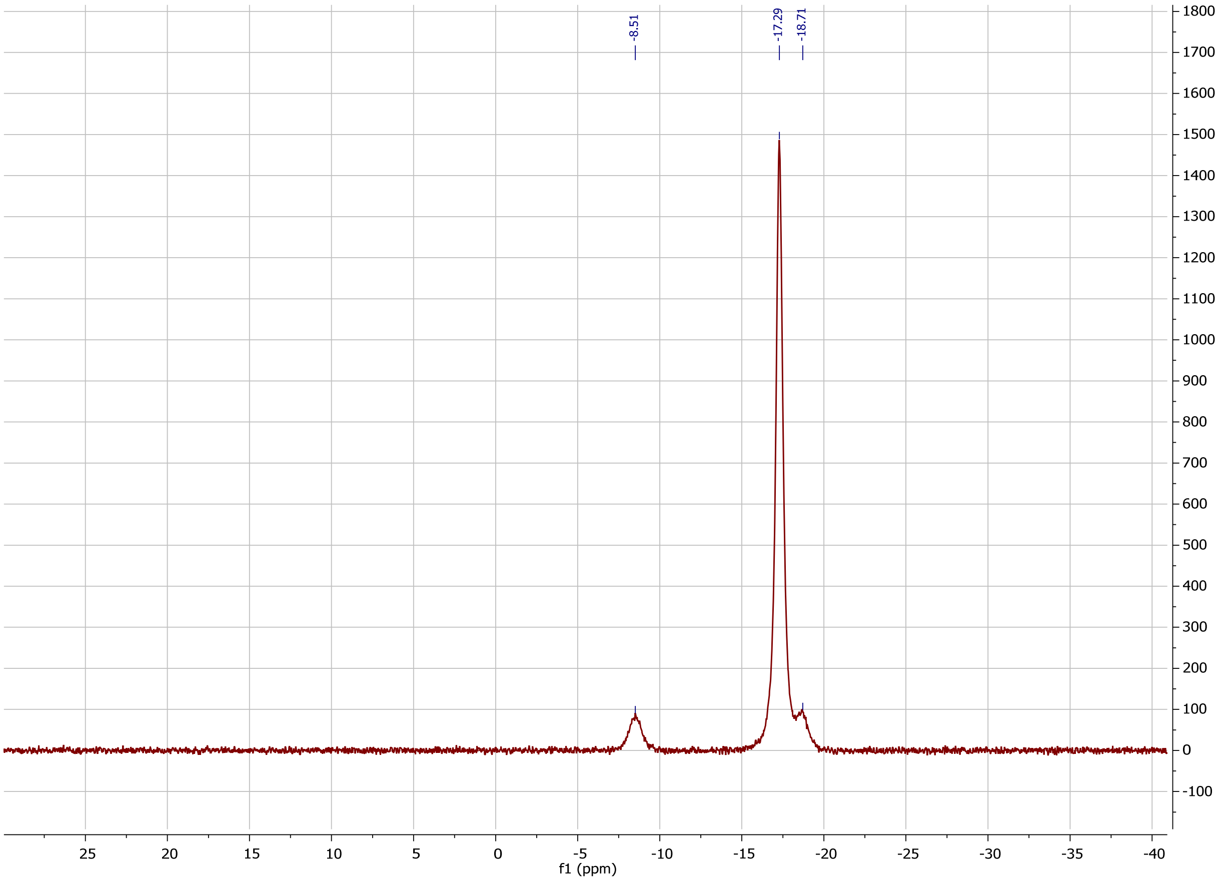

^{1}H NMR spectrum $(NBu_4)[B_{12}H_{11}(NHC(NHCH_3)CH_3)]$ 3b.

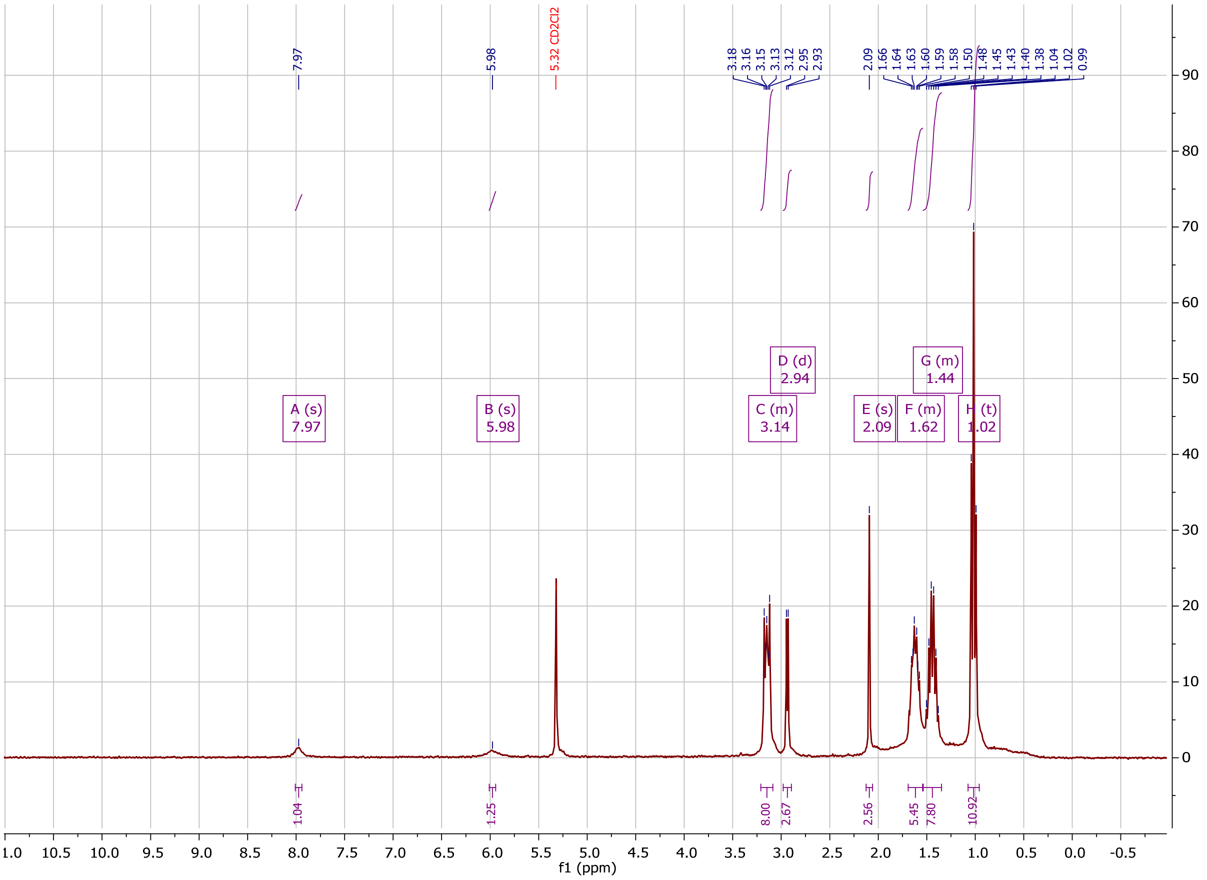

^{13}C NMR spectrum $(NBu_4)[B_{12}H_{11}(NHC(NHCH_3)CH_3)]$ 3b.

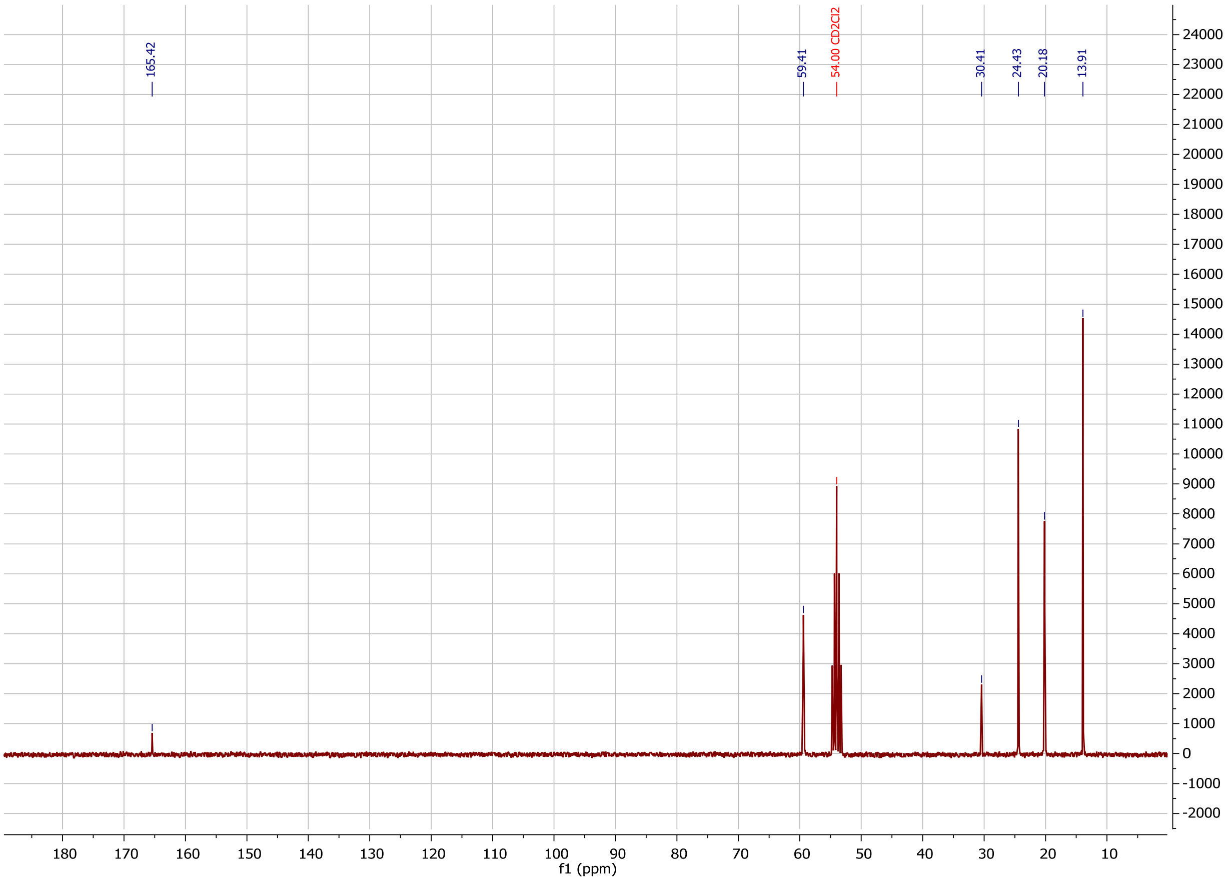

ESI-MS spectrum $(NBu_4)[B_{12}H_{11}(NHC(NHCH_3)CH_3)]$ (negative area) 3b.

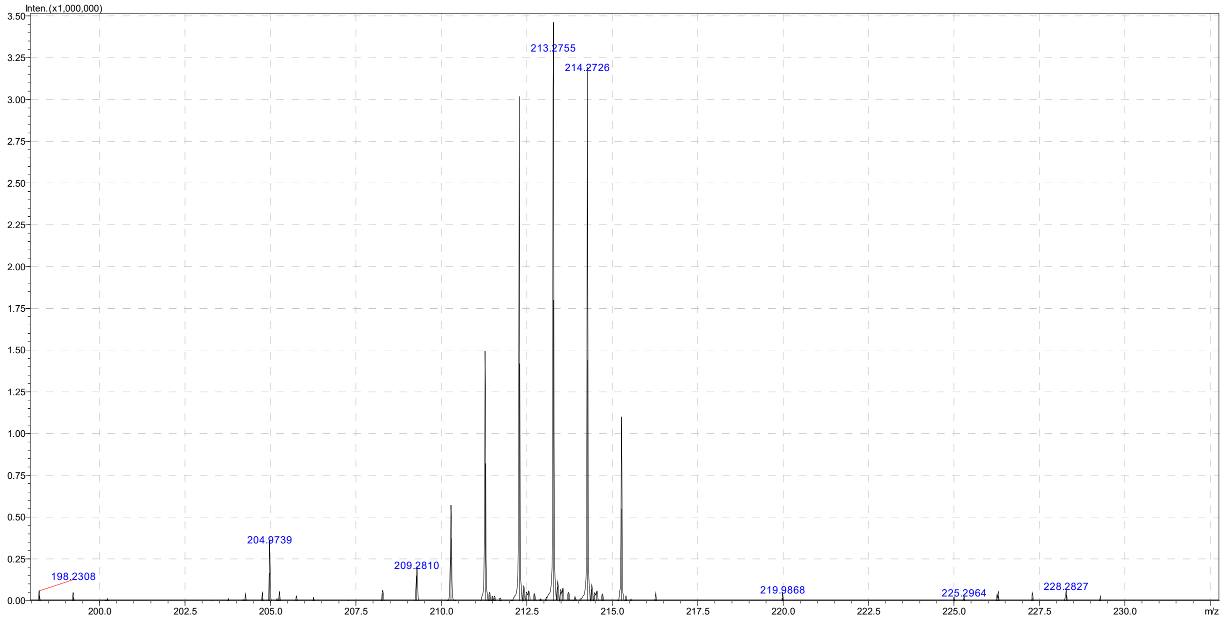

$^{11}B\{^1H\}$ NMR spectrum $(NBu_4)[B_{12}H_{11}(NHC(NHCH_2COOC_2H_5)CH_3)]$ 3c.

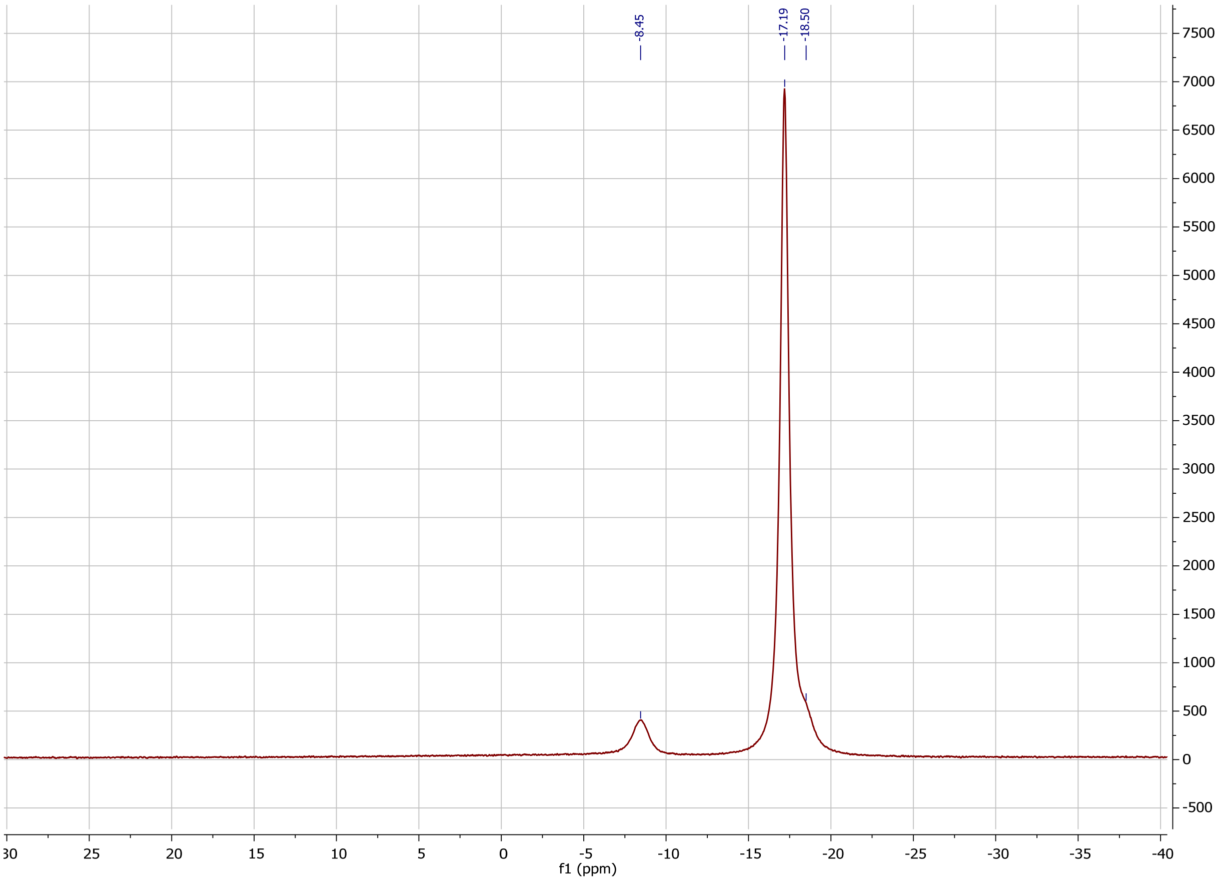

^1H NMR spectrum $(NBu_4)[B_{12}H_{11}(NHC(NHCH_2COOC_2H_5)CH_3)]$ 3c.

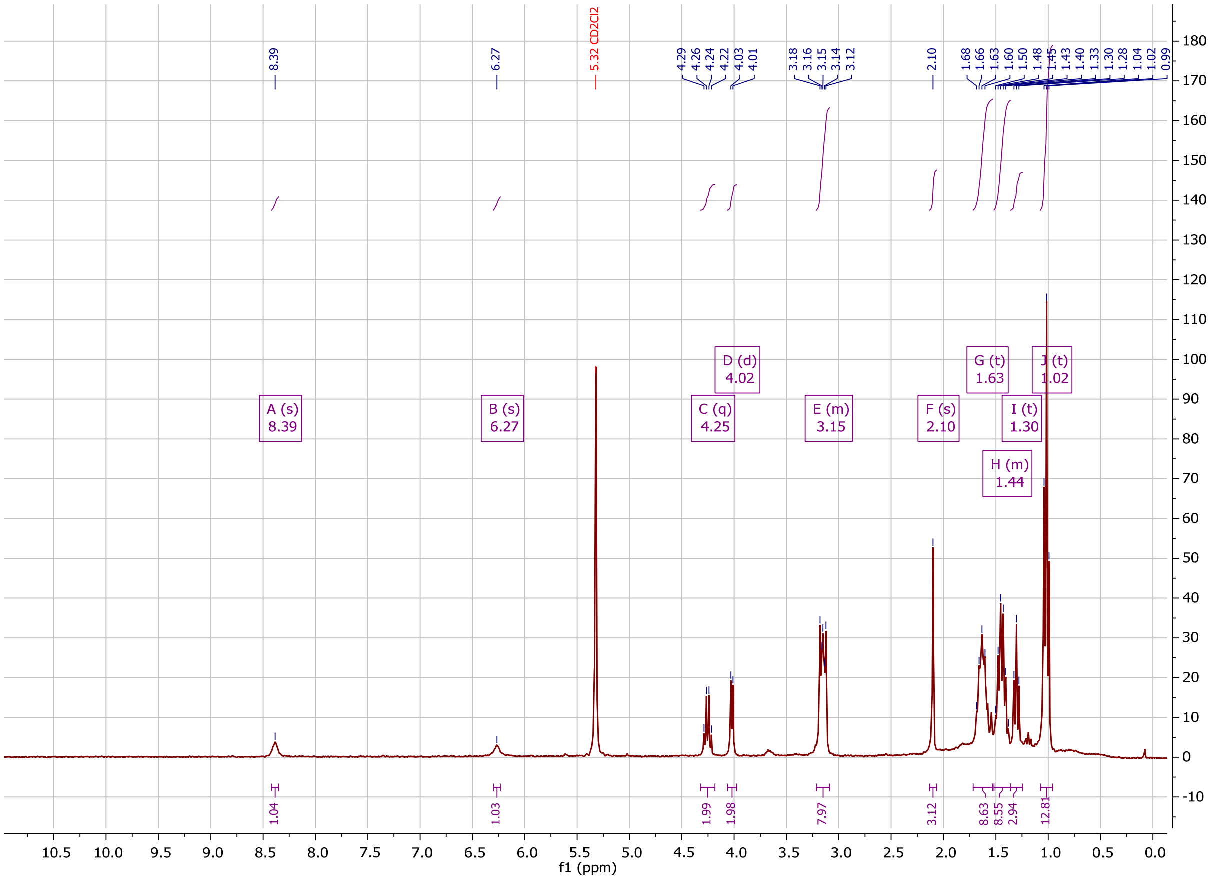

^{13}C NMR spectrum $(NBu_4)[B_{12}H_{11}(NHC(NHCH_2COOC_2H_5)CH_3)]$ 3c.

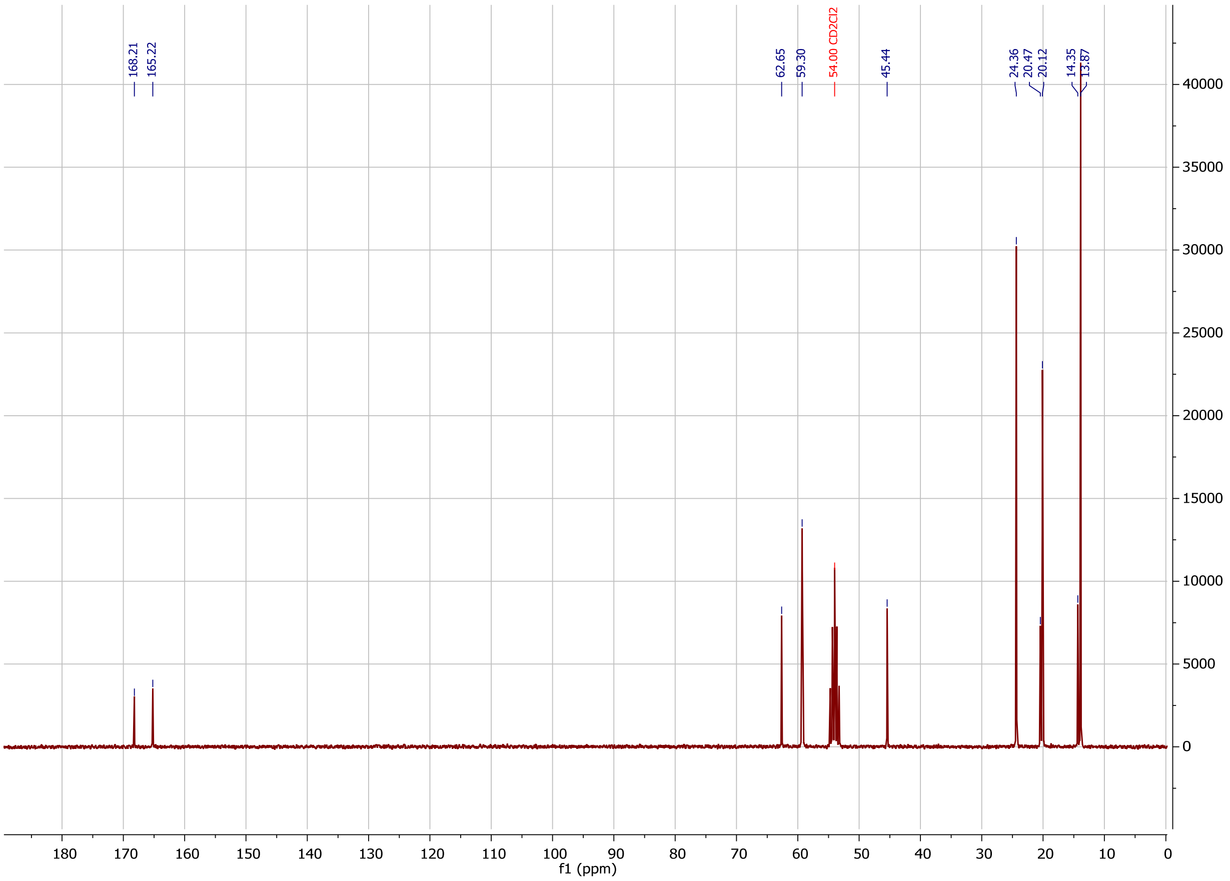

ESI-MS spectrum $(NBu_4)[B_{12}H_{11}(NHC(NHCH_2COOC_2H_5)CH_3)]$ (negative area) 3c.

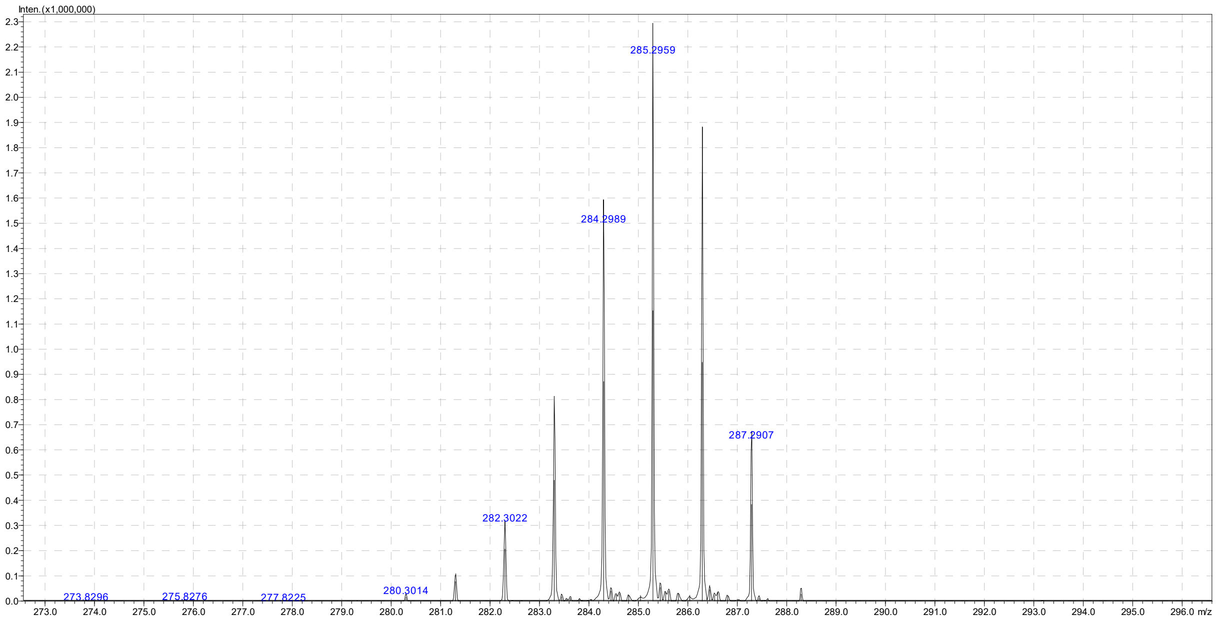

$^{11}B\{^{1}H\}$ NMR spectrum $(NBu_4)[B_{12}H_{11}(NHC(NHC_3H_7)CH_3)]$ 3d.

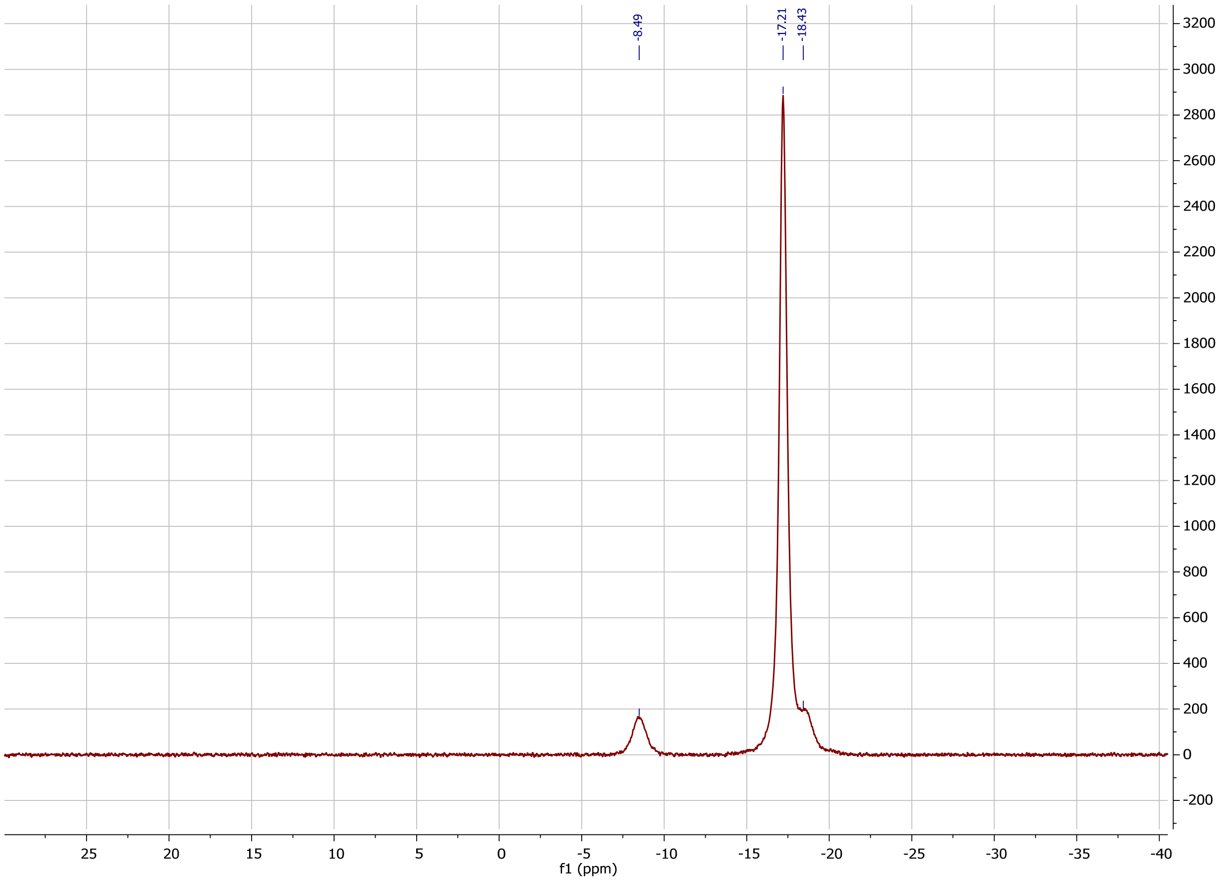

^{1}H NMR spectrum $(NBu_4)[B_{12}H_{11}(NHC(NHC_3H_7)CH_3)]$ 3d.

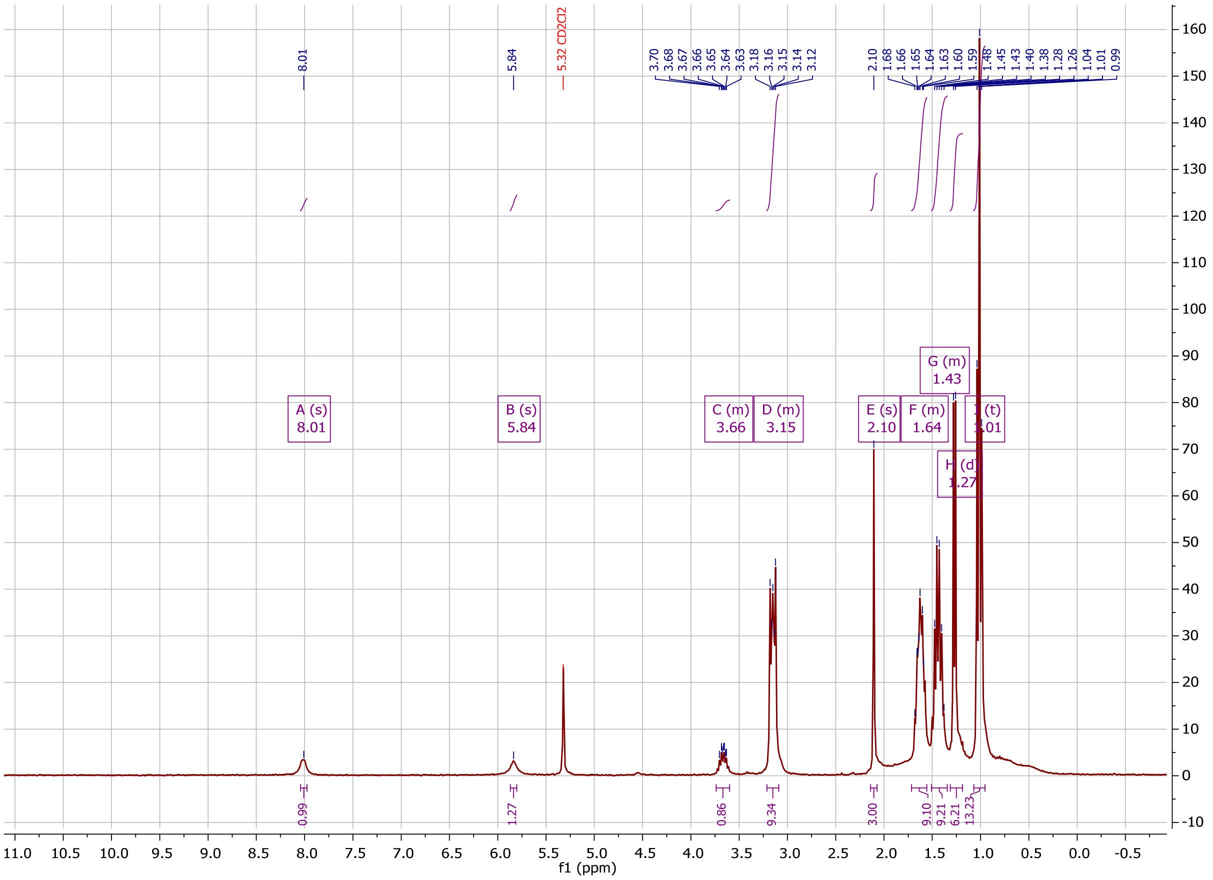

^{13}C NMR spectrum $(NBu_4)[B_{12}H_{11}(NHC(NHC_3H_7)CH_3)]$ 3d.

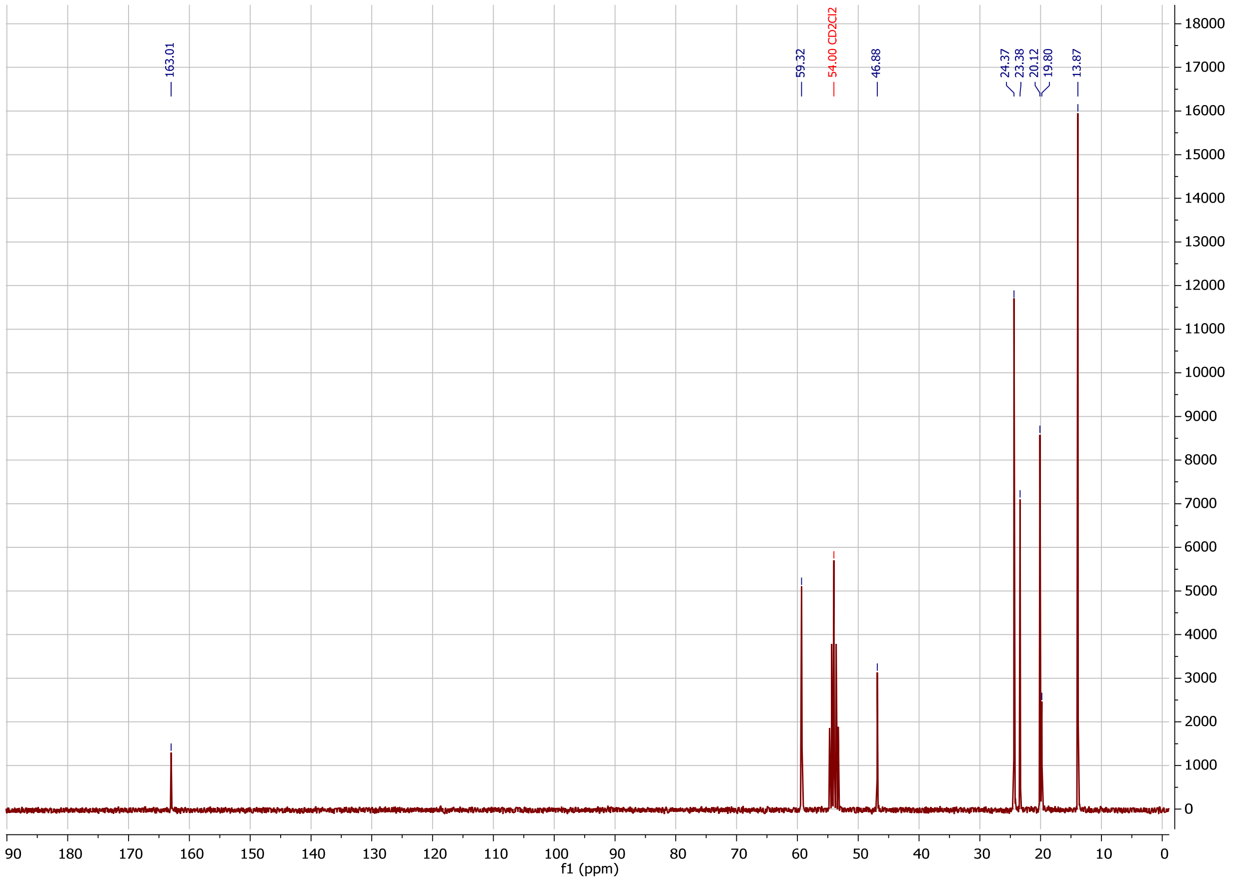

ESI-MS spectrum $(NBu_4)[B_{12}H_{11}(NHC(NHC_3H_7)CH_3)]$ (negative area) 3d.

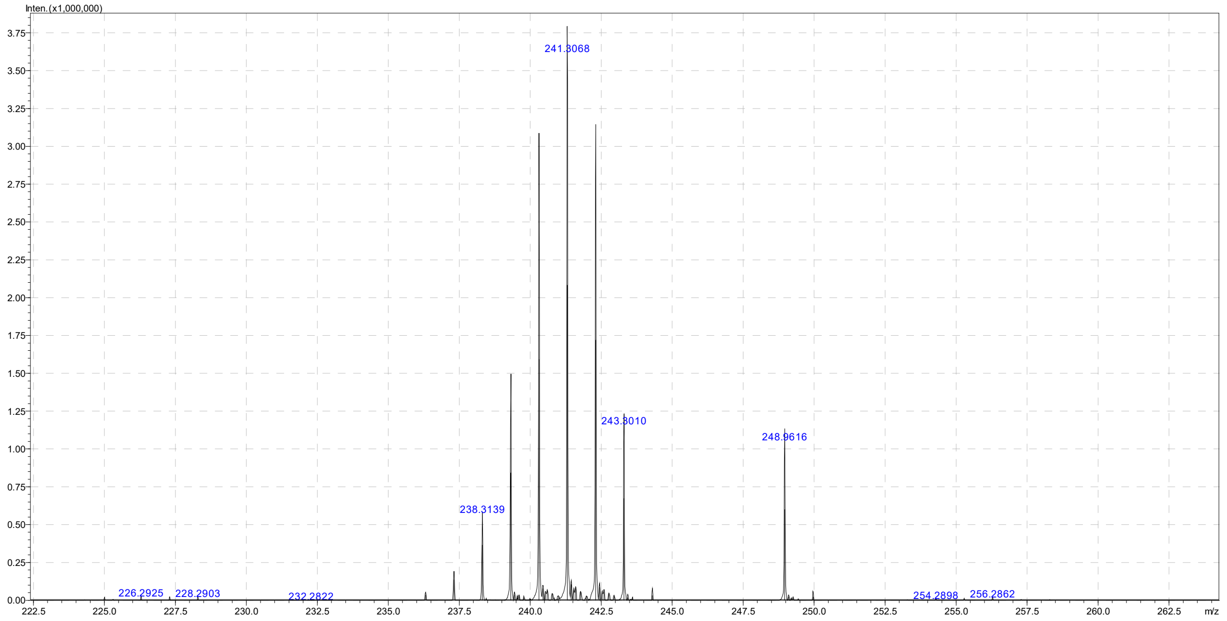

$^{11}B\{^1H\}$ NMR spectrum $(NBu_4)[B_{12}H_{11}(NHC(NHC_4H_9)CH_3)]$ 3e.

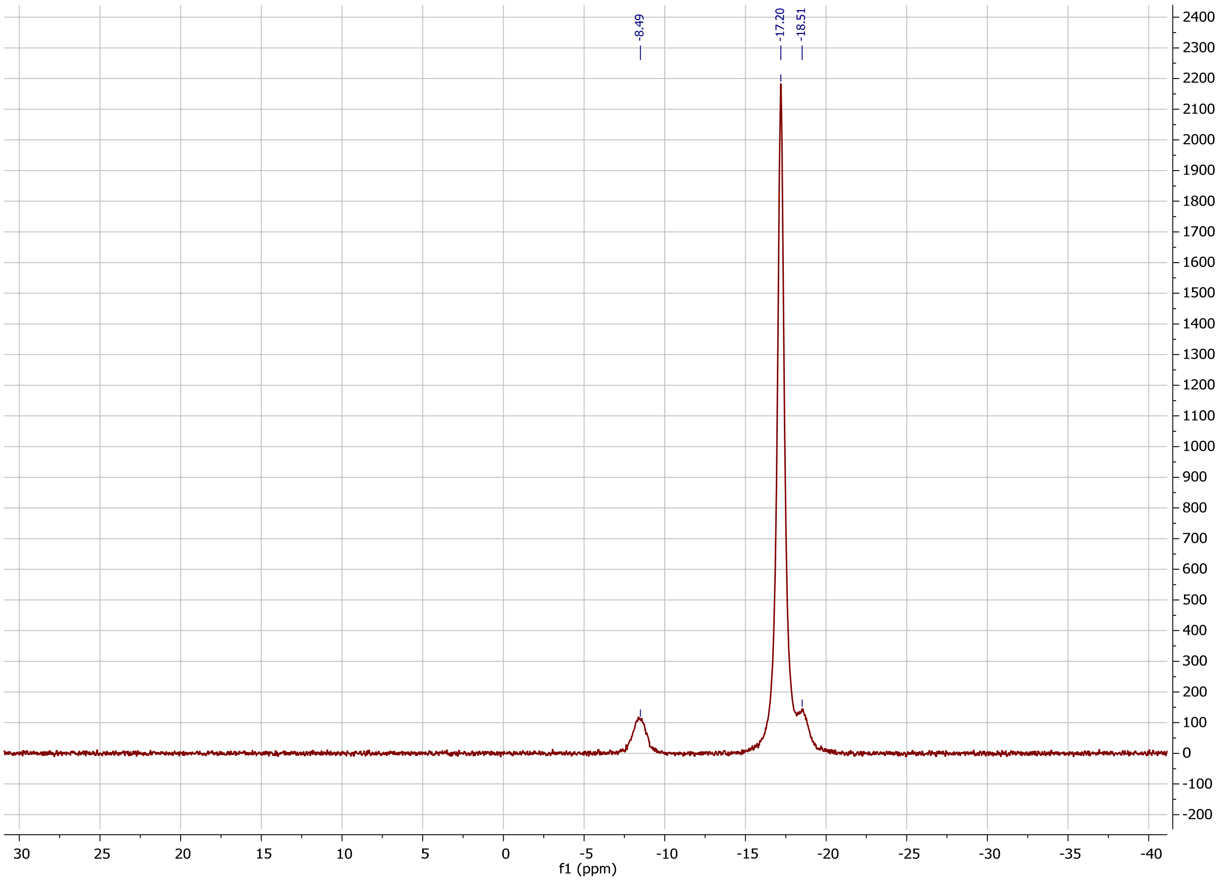

^1H NMR spectrum $(NBu_4)[B_{12}H_{11}(NHC(NHC_4H_9)CH_3)]$ 3e.

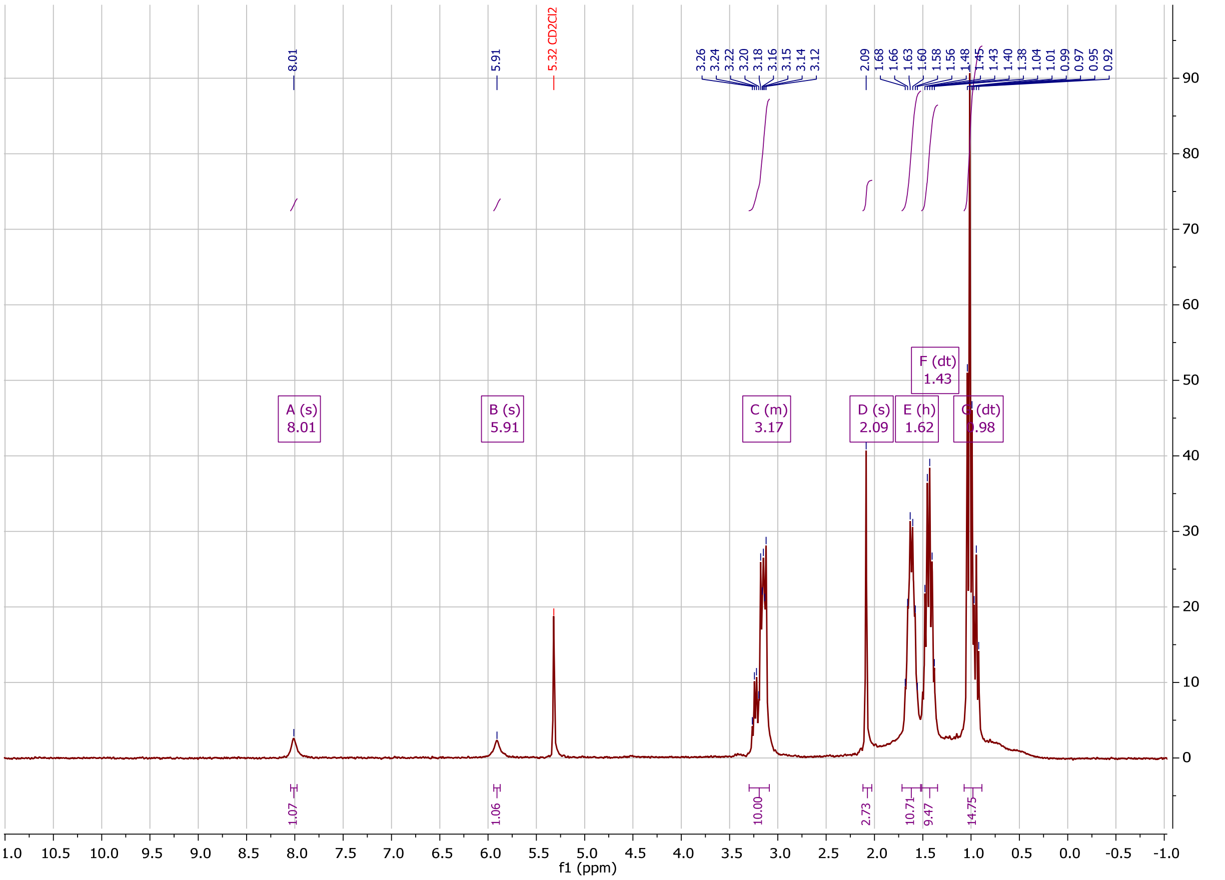

^{13}C NMR spectrum $(NBu_4)[B_{12}H_{11}(NHC(NHC_4H_9)CH_3)]$ 3e.

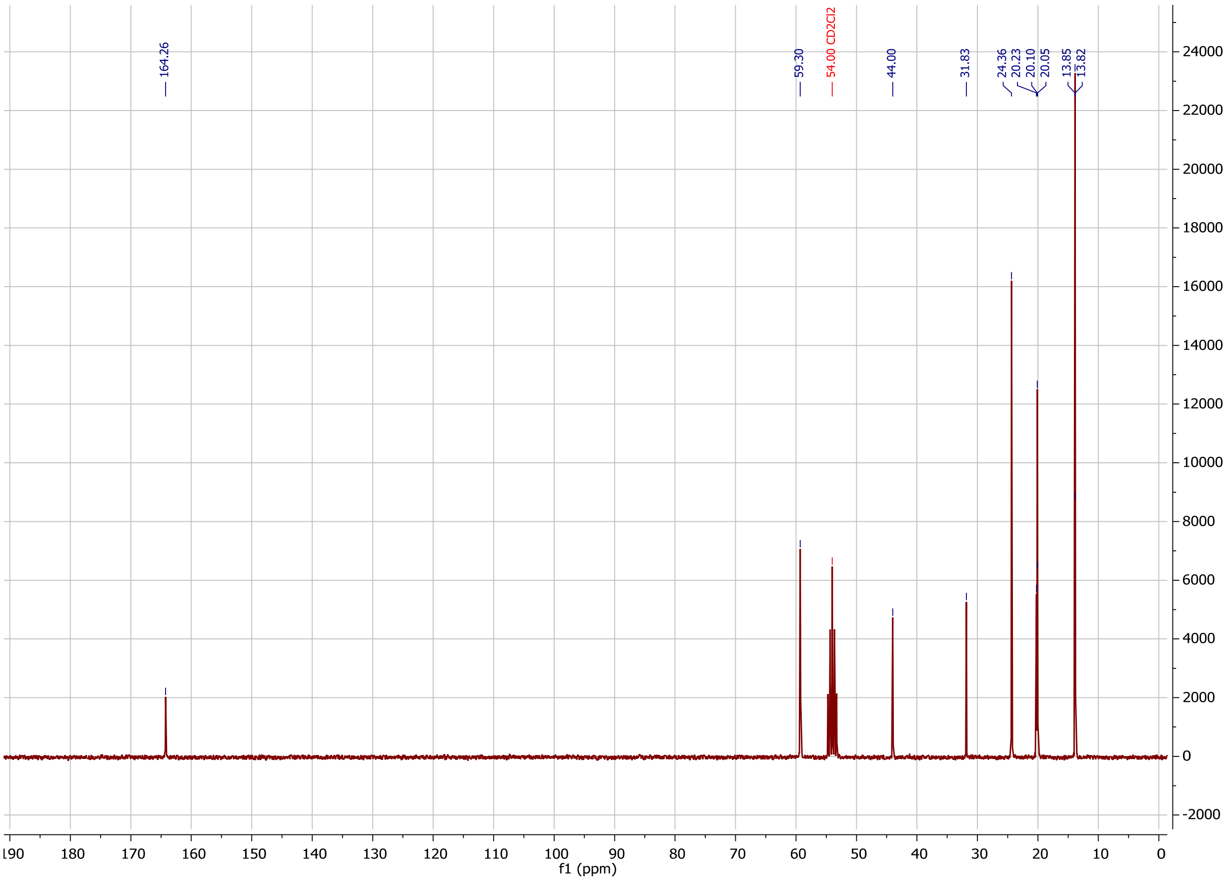

ESI-MS spectrum $(NBu_4)[B_{12}H_{11}(NHC(NHC_4H_9)CH_3)]$ (negative area) 3e.

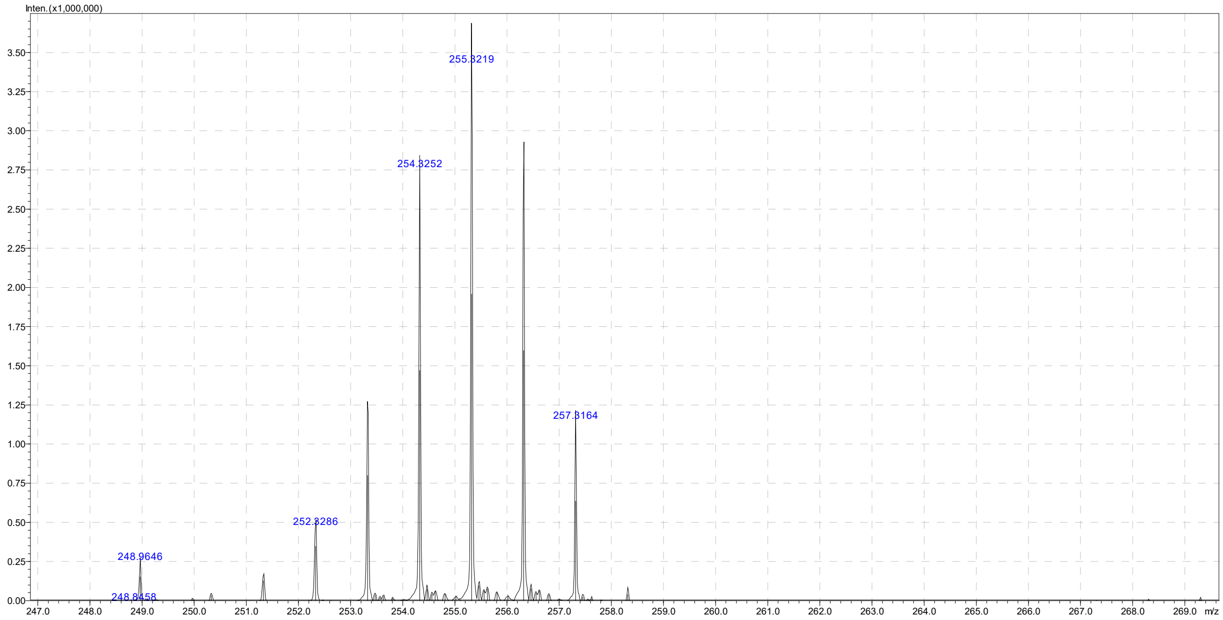

$^{11}B\{^{1}H\}$ NMR spectrum $(NBu_4)[B_{12}H_{11}(NHC(NH(CH_2)_2OH)CH_3)]$ 3f.

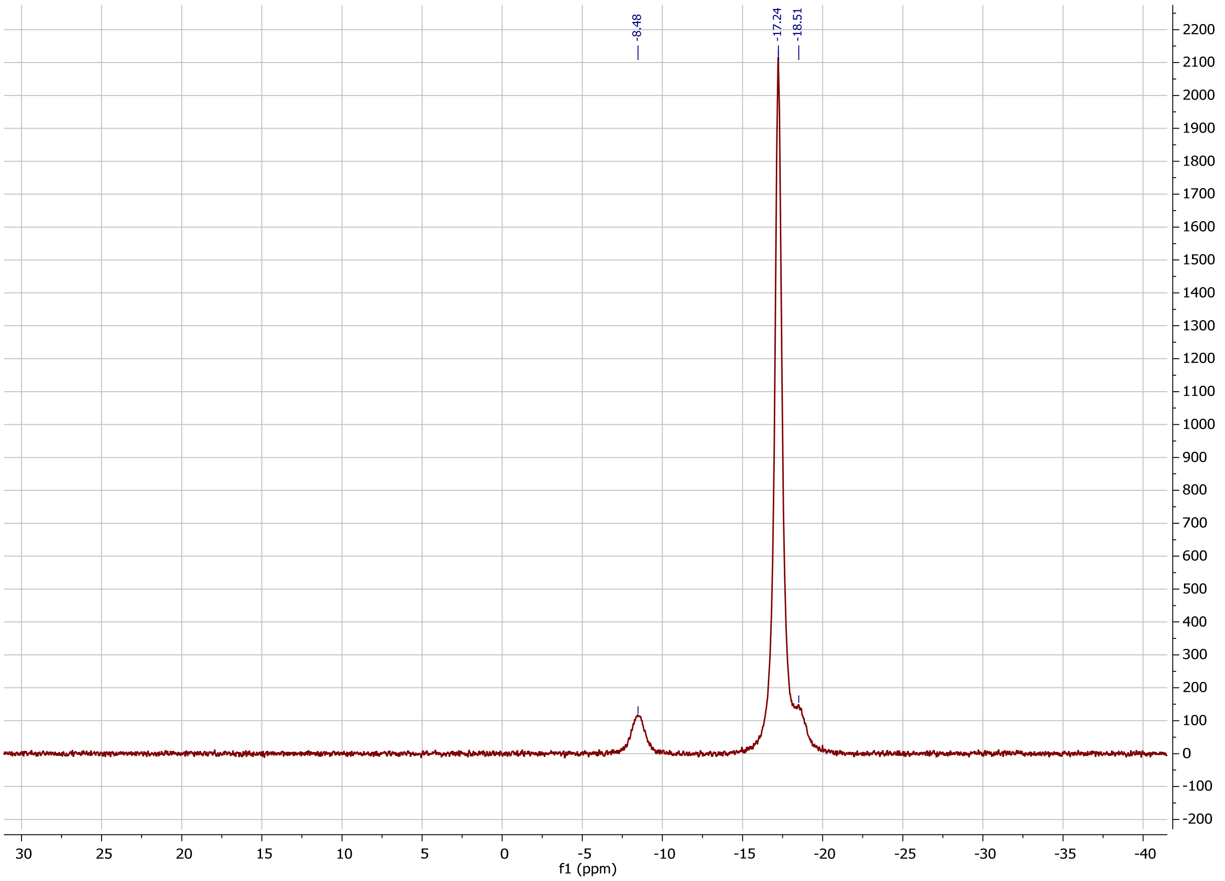

^{1}H NMR spectrum $(NBu_4)[B_{12}H_{11}(NHC(NH(CH_2)_2OH)CH_3)]$ 3f.

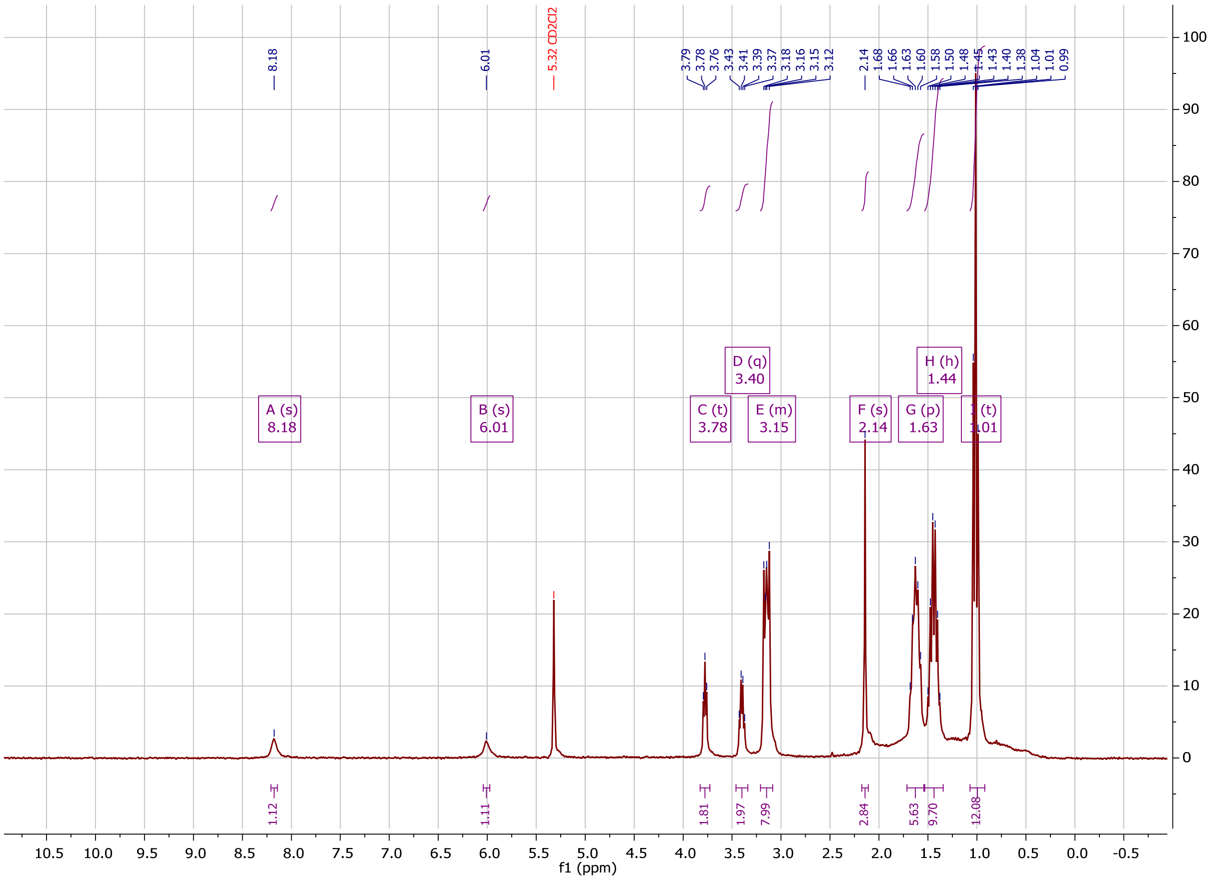

^{13}C NMR spectrum $(NBu_4)[B_{12}H_{11}(NHC(NH(CH_2)_2OH)CH_3)]$ 3f.

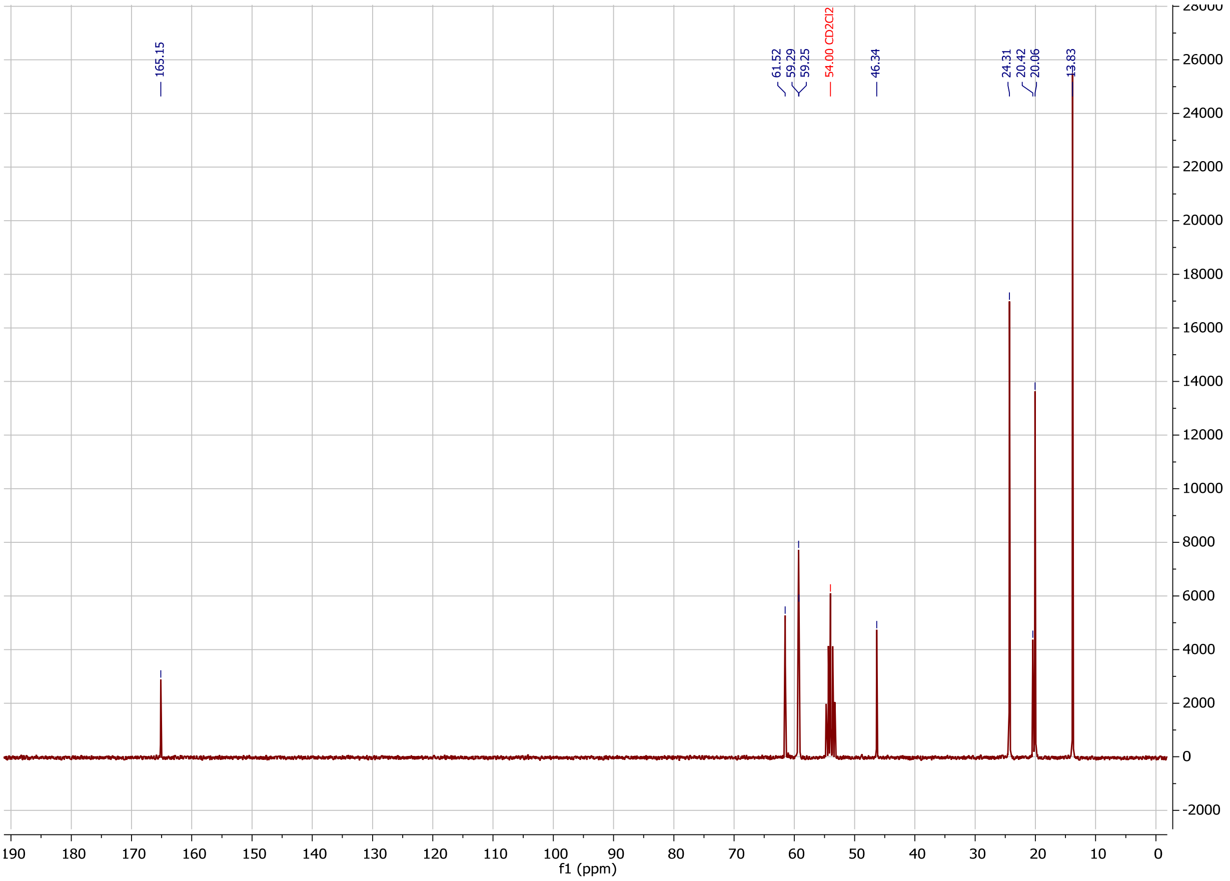

ESI-MS spectrum $(NBu_4)[B_{12}H_{11}(NHC(NH(CH_2)_2OH)CH_3)]$ (negative area) 3f.

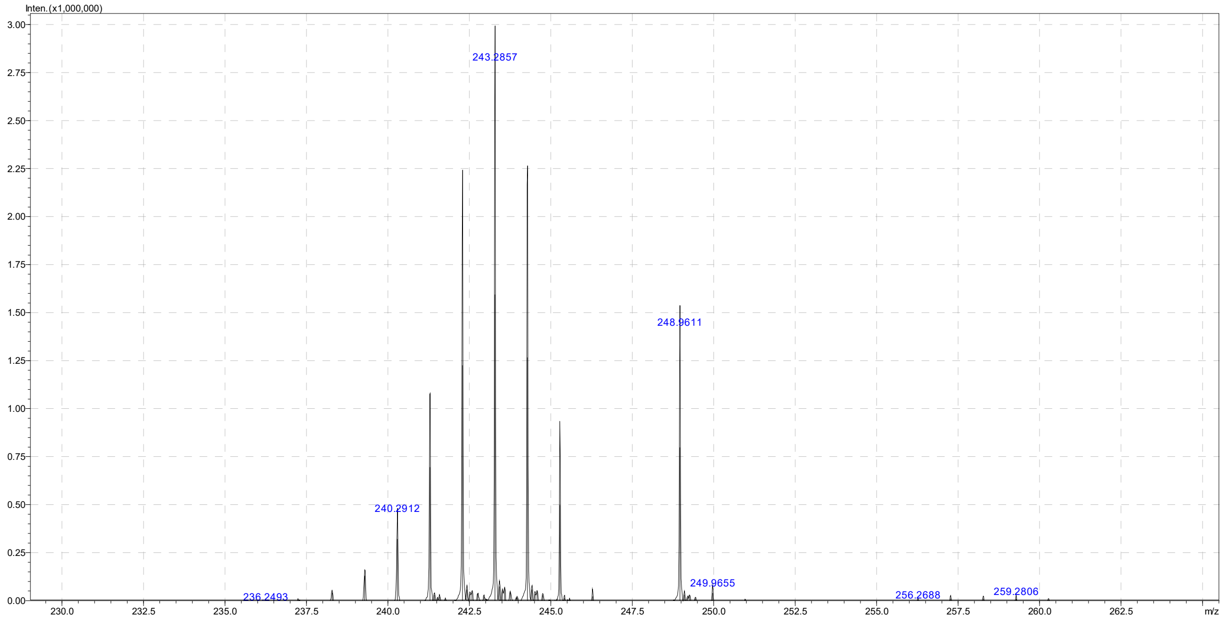

$^{11}B\{^{1}H\}$ NMR spectrum $(NBu_4)[B_{12}H_{11}(NHC(NHC_6H_5)CH_3)]$ 3g.

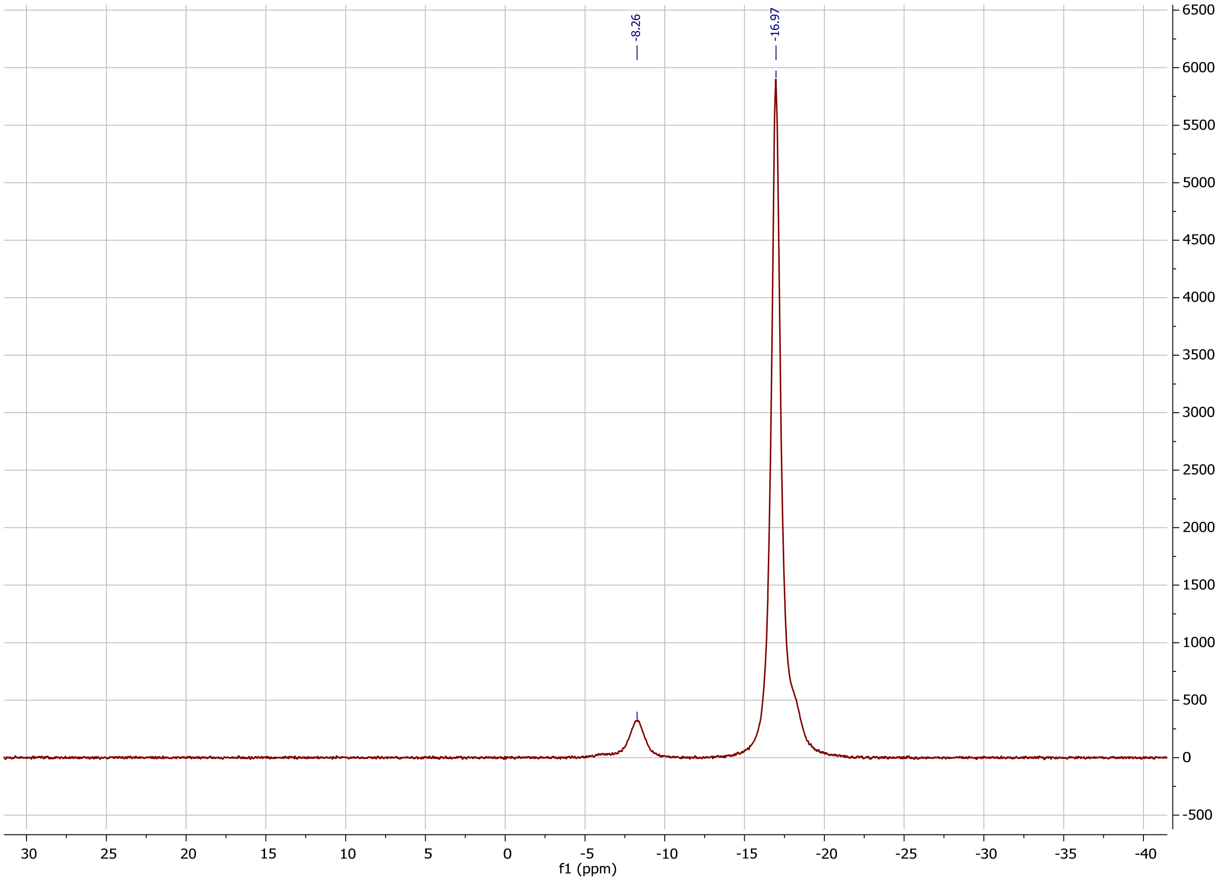

^{1}H NMR spectrum $(NBu_4)[B_{12}H_{11}(NHC(NHC_6H_5)CH_3)]$ 3g.

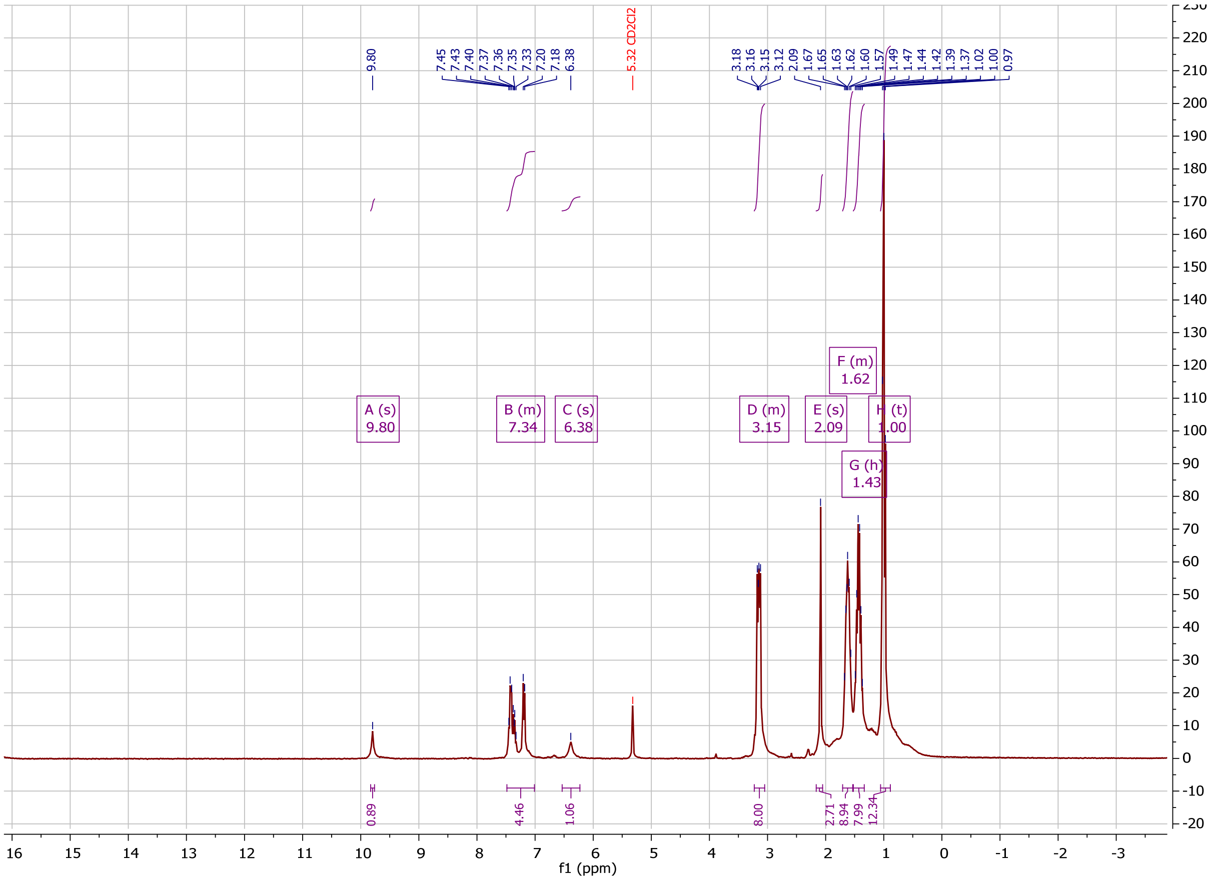

^{13}C NMR spectrum $(NBu_4)[B_{12}H_{11}(NHC(NHC_6H_5)CH_3)]$ 3g.

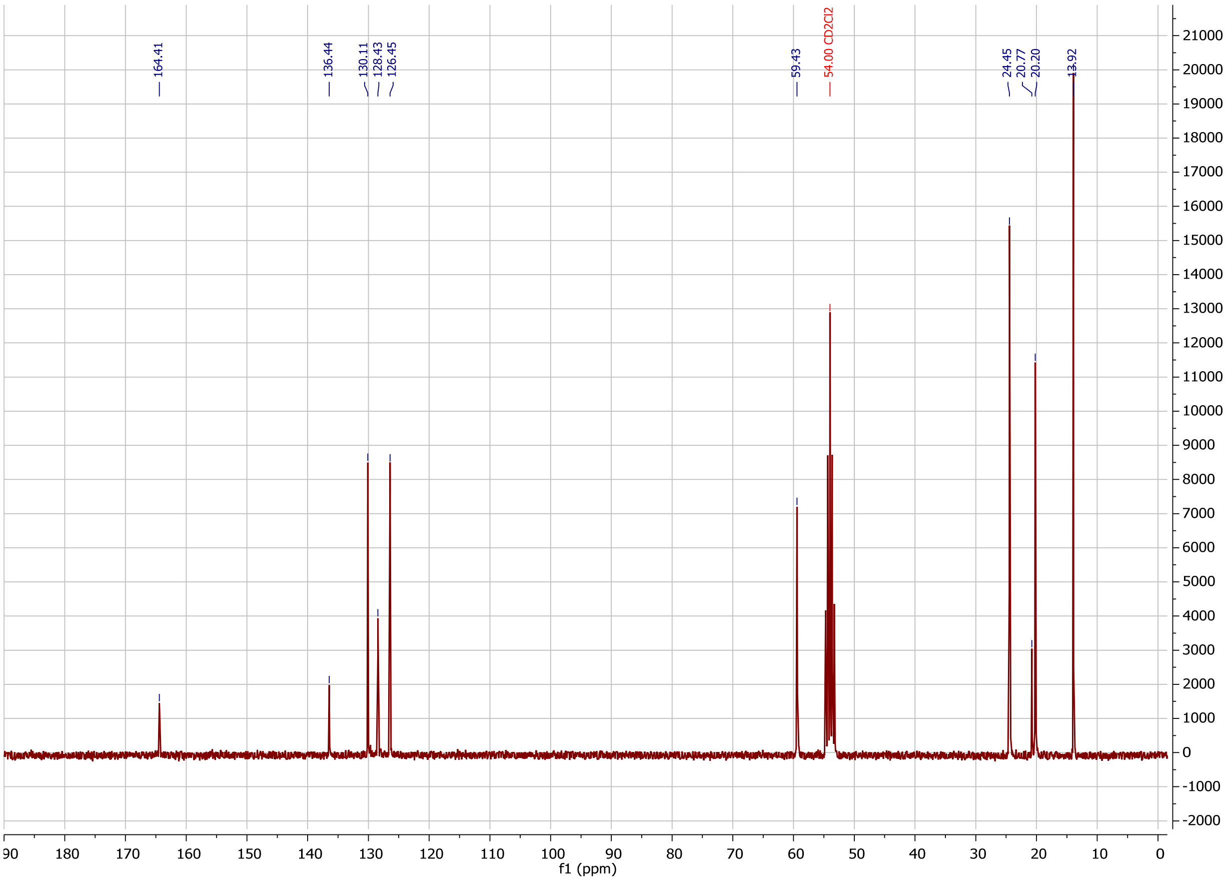

ESI-MS spectrum $(NBu_4)[B_{12}H_{11}(NHC(NHC_6H_5)CH_3)]$ (negative area) 3g.

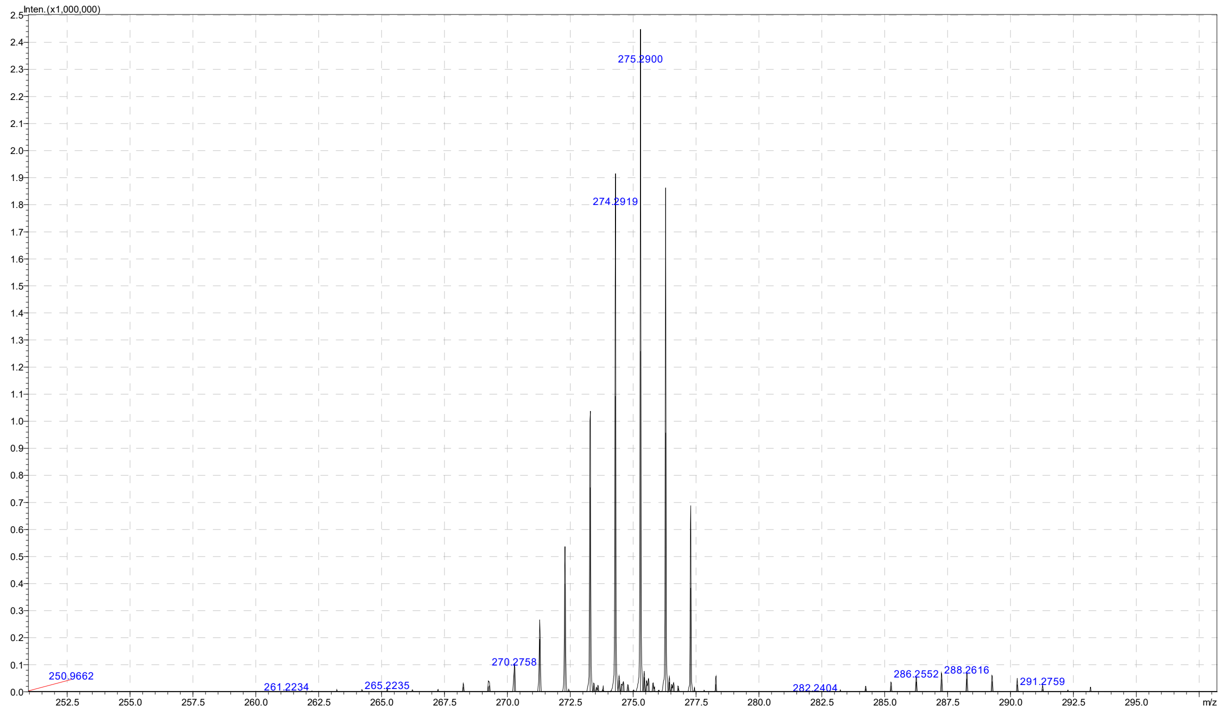

$^{11}B\{^{1}H\}$ NMR spectrum $(NBu_4)[B_{12}H_{11}(NHC(NHC_6H_{12})CH_3)]$ 3h.

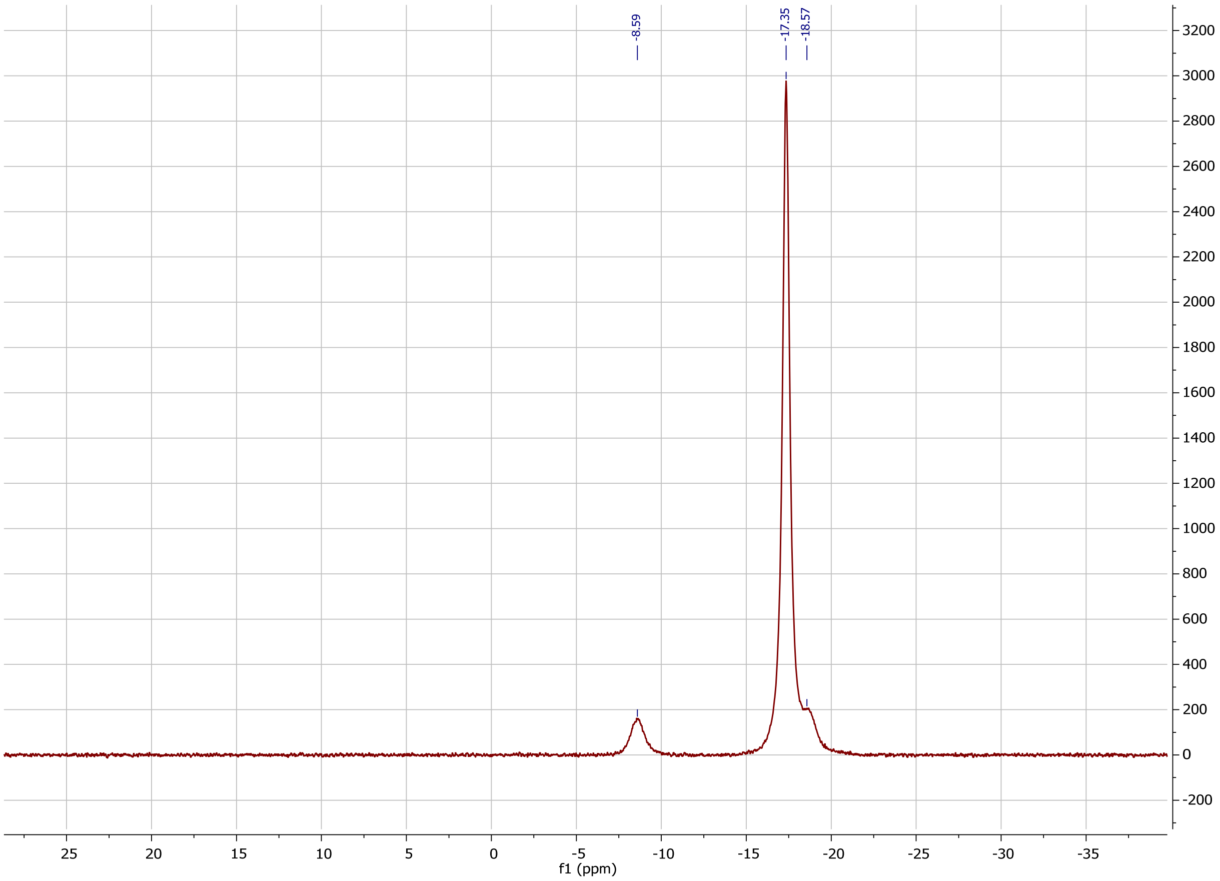

^{1}H NMR spectrum $(NBu_4)[B_{12}H_{11}(NHC(NHC_6H_{12})CH_3)]$ 3h.

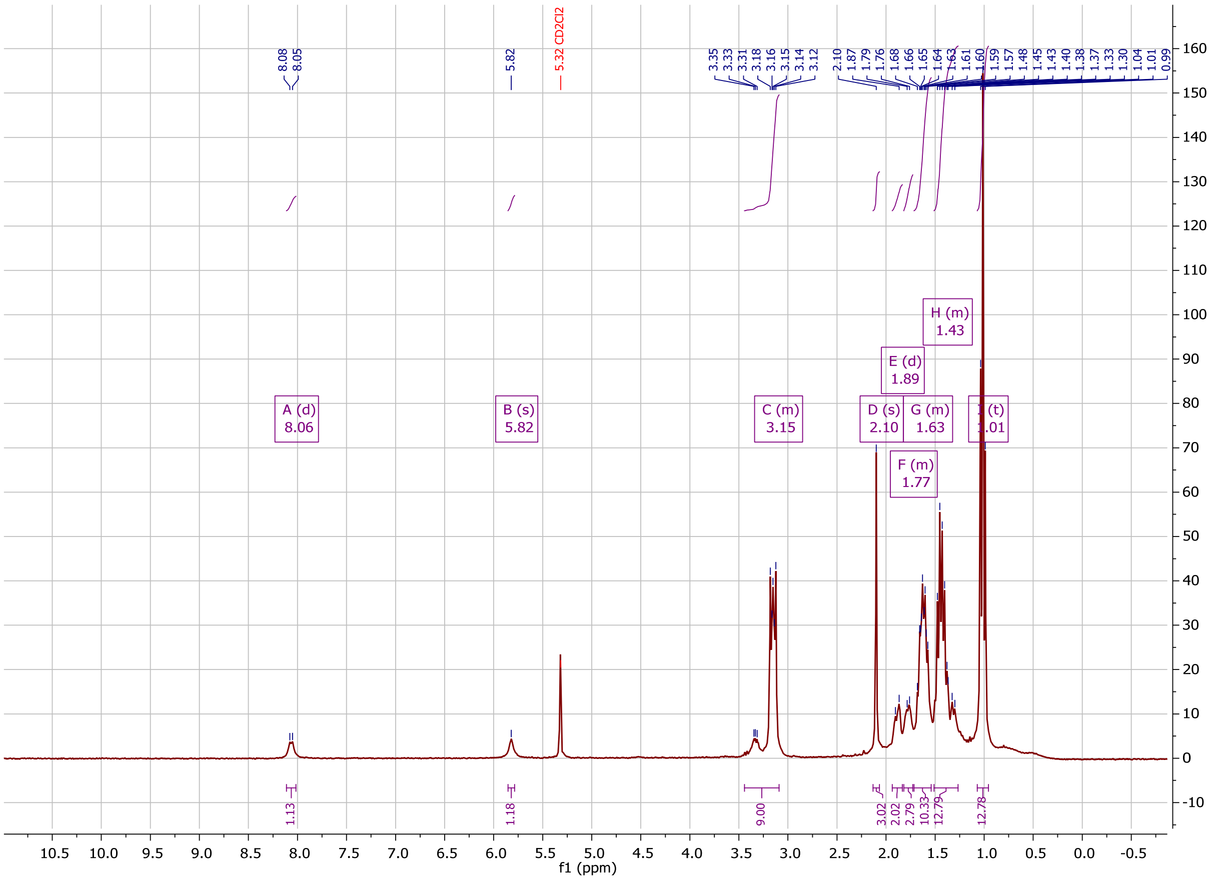

^{13}C NMR spectrum $(NBu_4)[B_{12}H_{11}(NHC(NHC_6H_{12})CH_3)]$ 3h.

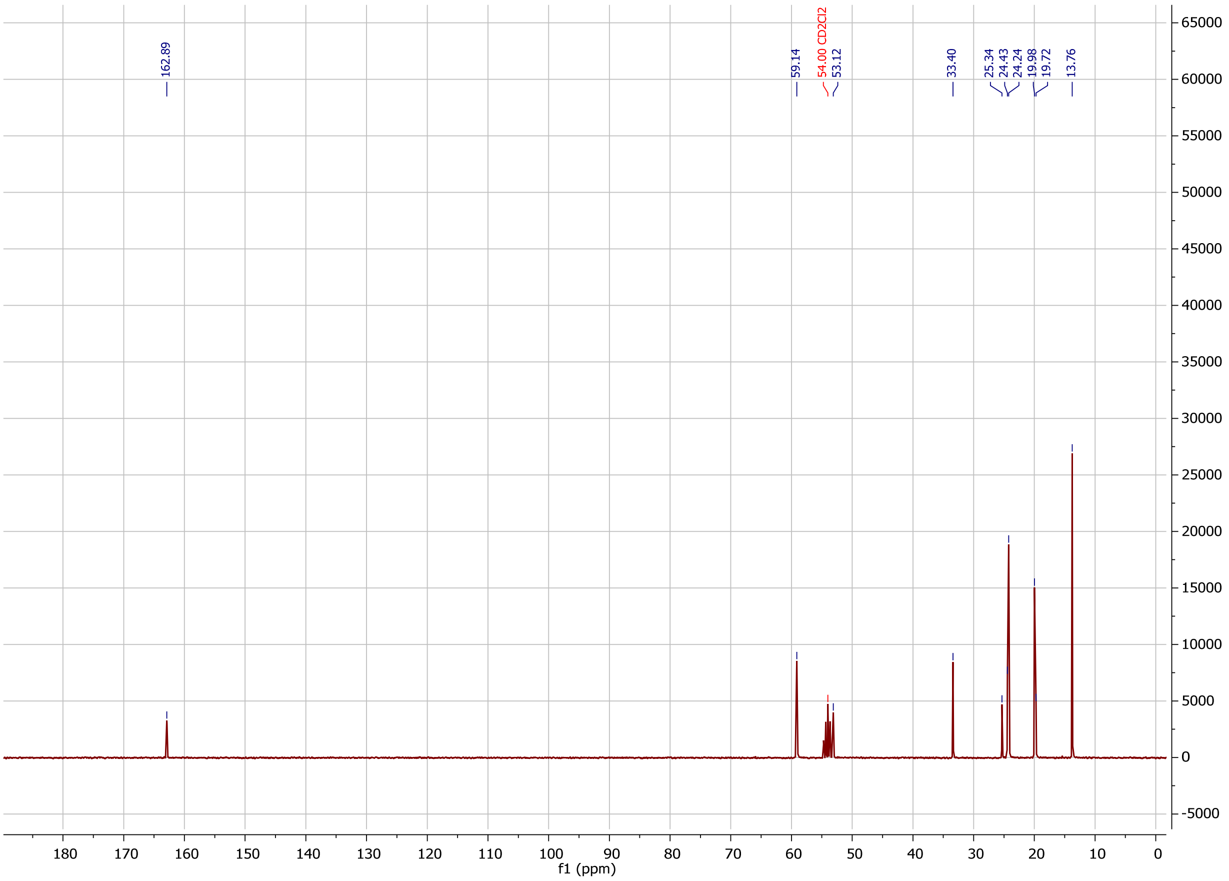

ESI-MS spectrum $(NBu_4)[B_{12}H_{11}(NHC(NHC_6H_{12})CH_3)]$ (negative area) 3h.

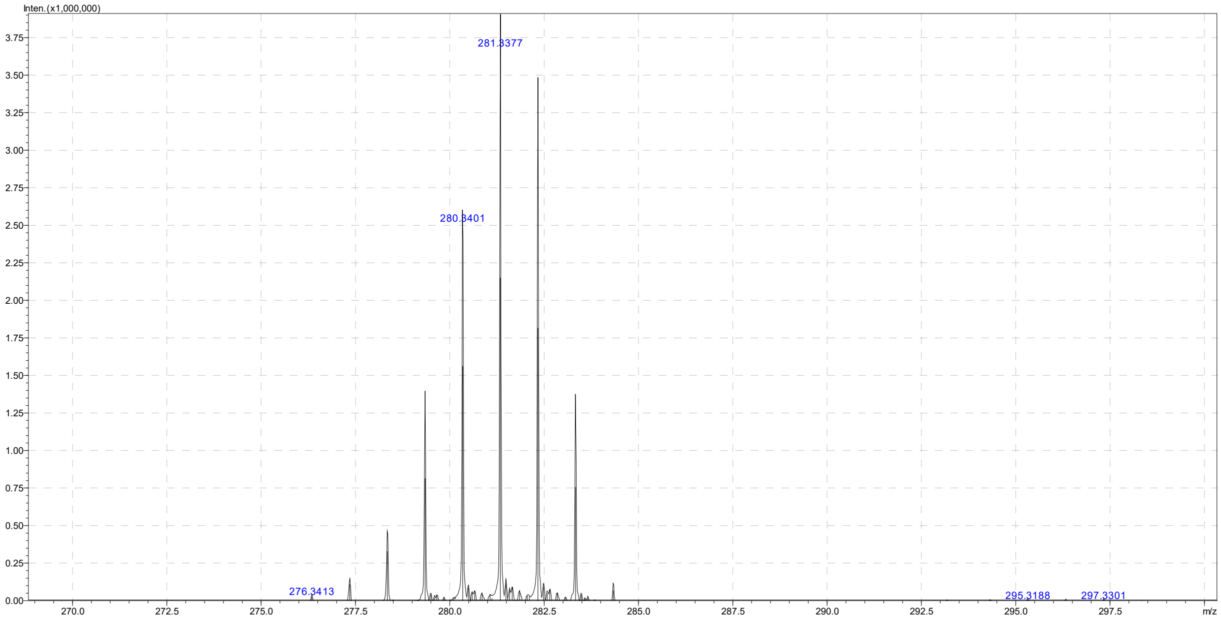

2. Computational details

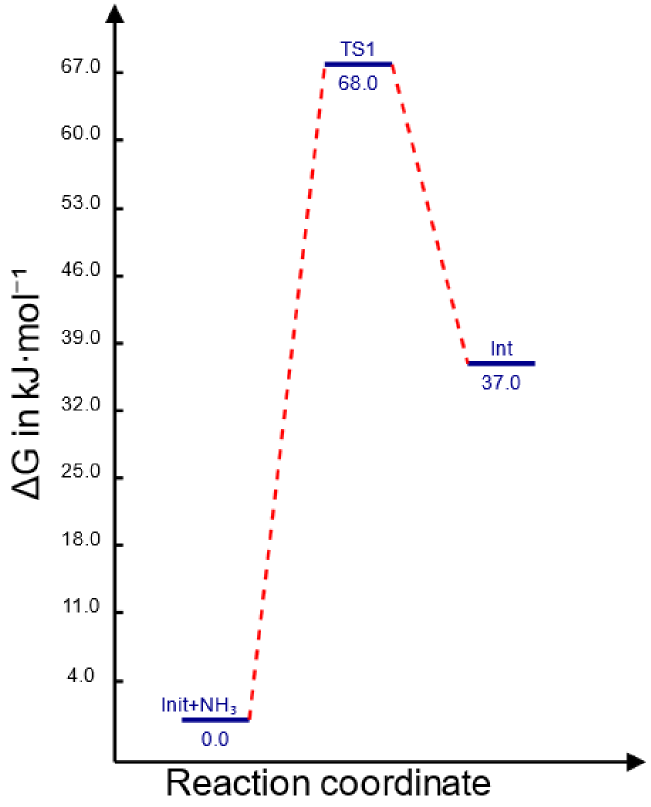

Figure S1. Energy profile of ammonia nucleophilic addition to nitrilium derivatives of *closo*-dodecaborate anion $[B_{12}H_{11}NCCH_3]^-$ with formation $[B_{12}H_{11}NCCH_3NH_3]^-$ (Int) in tetrahydrofuran solution.

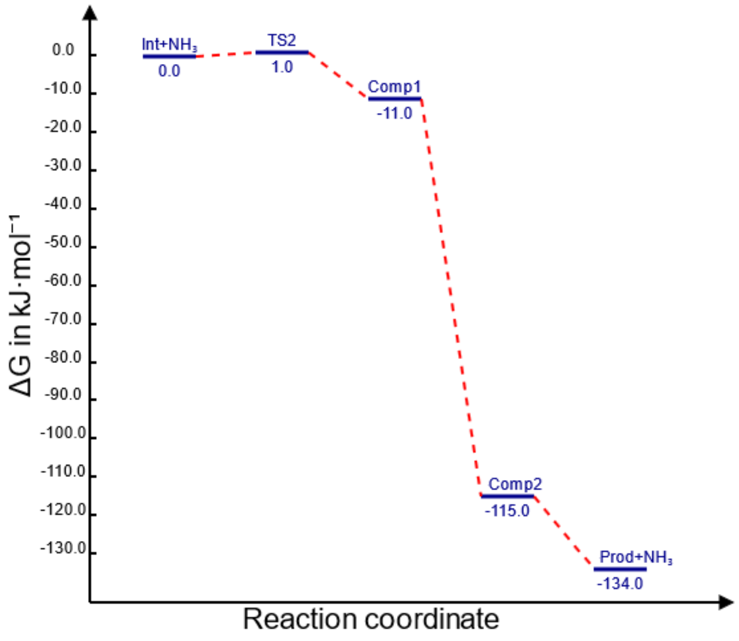

Figure S2. Energy profile of isomerisation of $[B_{12}H_{11}NCCH_3NH_3]^-$ (Int) to $[B_{12}H_{11}NHCCH_3NH_2]^-$ (Prod) in tetrahydrofuran solution.

Table S1. Main parameters of non-covalent intermolecular dihydrogen H–H interaction in $[B_{12}H_{11}NHCCH_3NH_2]^-$: bond lengths, Wiberg index, and main topological parameters of electron density for B–C interactions. $\rho(r)$ – electron density at the bcp, $\nabla^2\rho(r)$ – Laplacian of electron density at the bcp, H_b – total energy at the bcp, δ(B–C) – delocalisation index, ε_b – ellipticity at the bcp.

H–H Length (Å)	Wiberg Index	$\rho(r)$ (e Å^{-3})	$\nabla^2\rho(r)$ (e Å^{-5})	H_b (h e^{-1})	δ(B–H)	ε_b
1.66	0.02	0.026	0.060	-0.007	0.782	0.075

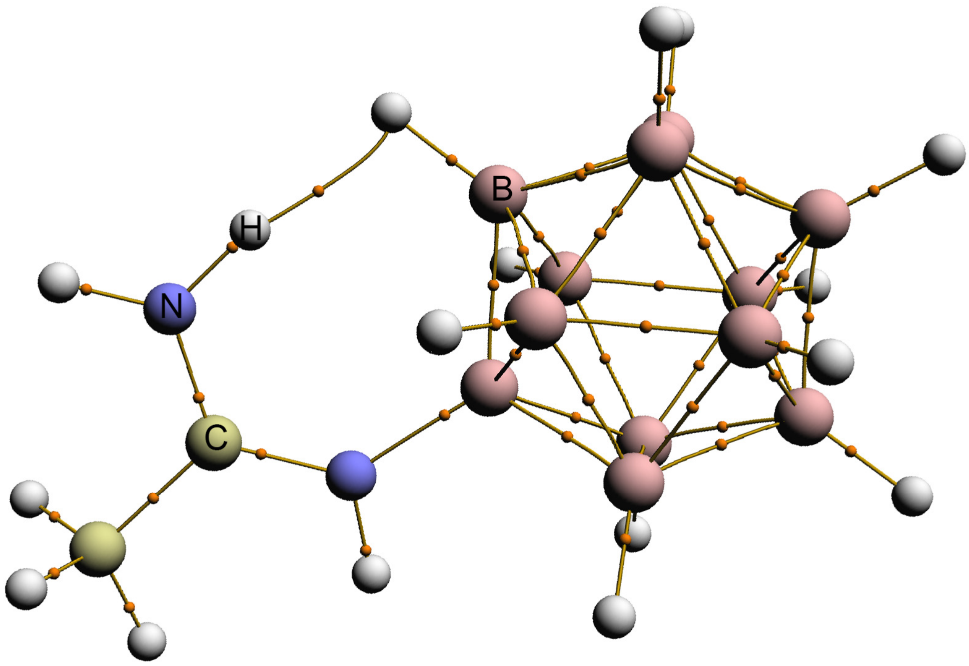

Figure S3. Molecular graphs for $[B_{12}H_{11}NHCCH_3NH_2]^-$ anion. B–All bond critical points are indicated by small orange circles.

Table S2. Cartesian atomic coordinates of the calculated optimised equilibrium model structures. All coordinates are given in Angstrom units.

Gas Phase:

Compound	Atom	x	y	z
$[B_{12}H_{11}NHCCH_3NH_2]^-$	N	4.329892	13.546046	2.147225
	H	4.252686	13.308663	1.167292
	N	3.245166	13.649785	4.162364
	H	4.086469	14.046572	4.591482
	C	3.25947	13.362999	2.85905
	C	2.003253	12.80527	2.251699
	H	2.093717	12.726471	1.167202
	H	1.148478	13.445821	2.485761
	H	1.810703	11.807254	2.656689
	B	6.809825	14.570724	1.317888
	H	6.396126	14.668846	0.1889
	B	5.742389	13.984592	2.588393
	B	7.074309	12.960589	2.051661

	H	6.845839	11.954761	1.428414
	B	8.452983	14.035731	1.732458
	H	9.260057	13.77892	0.881534
	B	7.939154	15.715323	2.069893
	H	8.381238	16.65105	1.461669
	B	6.237	15.684184	2.595908
	H	5.443729	16.555924	2.355955
	B	6.162378	14.755983	4.122893
	H	5.360504	15.031264	4.98879
	B	6.666015	13.069948	3.79047
	H	6.169783	12.142486	4.37795
	B	8.36583	13.112668	3.259795
	H	9.110038	12.201464	3.497924
	B	8.900945	14.814171	3.272139
	H	10.035542	15.113373	3.525467
	B	7.535975	15.829789	3.804894
	H	7.689539	16.840375	4.434338
	B	7.798968	14.218458	4.540557

Water Solution:

Compound	Atom	x	y	z
$[B_{12}H_{11}NCCH_3]^-$ **Init**	N	4.27857	13.825562	2.008663
	C	3.192122	13.588097	1.724496
	C	1.827647	13.291283	1.36682
	H	1.815177	12.846079	0.370157
	H	1.251941	14.219052	1.371584
	H	1.417491	12.590826	2.097168
	B	6.83808	14.714404	1.159719
	H	6.496149	14.874442	0.022491
	B	5.70478	14.133825	2.382026
	B	7.010441	13.072051	1.853454
	H	6.789391	12.093697	1.198479
	B	8.439797	14.103231	1.626902
	H	9.273766	13.841437	0.805365
	B	7.979797	15.784969	2.000005
	H	8.485528	16.719425	1.442701
	B	6.263352	15.805182	2.459025
	H	5.527399	16.718861	2.217642
	B	6.080008	14.838362	3.955461
	H	5.216742	15.084492	4.748741
	B	6.542827	13.148891	3.581114
	H	6.001074	12.223166	4.115133
	B	8.25738	13.141269	3.116168
	H	8.961058	12.198538	3.350057
	B	8.858228	14.81403	3.204704
	H	9.994597	15.059434	3.503049
	B	7.513707	15.861345	3.719044
	H	7.69272	16.848407	4.376707
	B	7.685573	14.227174	4.409419
	H	7.986495	14.053066	5.557808
**NH_3**	N	N	2.053076	23.982834
	H	H	2.053144	24.923379
	H	H	2.867812	23.512607
	H	H	1.238387	23.512556
$[B_{12}H_{11}NCCH_3*NH_3]^-$	N	4.109332	13.99683	2.453639

**TS1**	C	3.208373	13.645886	1.786348
	C	1.767798	13.465072	1.603273
	H	1.433899	14.042019	0.739017
	H	1.262023	13.81383	2.505866
	H	1.547988	12.40976	1.433108
	B	6.660539	14.771388	1.457281
	H	6.260986	14.948349	0.339089
	B	5.559768	14.240367	2.744028
	B	6.805402	13.131035	2.141421
	H	6.510494	12.159249	1.501388
	B	8.263002	14.096861	1.823872
	H	9.033562	13.799671	0.952925
	B	7.894949	15.794807	2.218445
	H	8.403935	16.708466	1.628738
	B	6.211154	15.88358	2.784794
	H	5.508472	16.835115	2.585719
	B	6.084024	14.931401	4.288831
	H	5.289077	15.217899	5.139883
	B	6.445405	13.229198	3.891063
	H	5.907461	12.322997	4.464233
	B	8.128746	13.144252	3.323192
	H	8.804343	12.171057	3.517665
	B	8.807242	14.789781	3.369732
	H	9.970076	14.986998	3.596662
	B	7.542388	15.891939	3.962828
	H	7.80464	16.87189	4.60436
	B	7.686732	14.254432	4.645774
	H	8.053212	14.066742	5.773503
	N	3.889351	12.923745	-0.047466
	H	4.366605	13.695677	-0.505668
	H	3.209245	12.534587	-0.694779
	H	4.584005	12.21124	0.161174
$[B_{12}H_{11}NCCH_3NH_3]^-$	N	3.949068	14.108182	2.62632
**Int**	C	3.289415	13.624935	1.681534
	C	1.804236	13.528031	1.569705
	H	1.454666	14.010362	0.65064
	H	1.342702	14.012311	2.42963
	H	1.491068	12.479111	1.535277
	B	6.539763	14.795898	1.469171
	H	6.117137	15.001561	0.362814
	B	5.442871	14.260941	2.77655
	B	6.682288	13.148782	2.122874
	H	6.384857	12.17507	1.479086
	B	8.144362	14.088141	1.763782
	H	8.874773	13.789057	0.859328
	B	7.820183	15.783127	2.19902
	H	8.324888	16.697178	1.605269
	B	6.158415	15.888253	2.827036
	H	5.474586	16.862818	2.669885
	B	6.071569	14.917588	4.313119
	H	5.31706	15.206018	5.201248
	B	6.378082	13.221793	3.877222
	H	5.85233	12.311376	4.457327
	B	8.043361	13.111729	3.251654
	H	8.704317	12.120548	3.402825
	B	8.756804	14.74233	3.298812

	H	9.931302	14.912814	3.485661
	B	7.534742	15.855842	3.954911
	H	июл.85	16.820569	4.602151
	B	7.670591	14.206921	4.604874
	H	8.073524	13.991671	5.715658
	N	3.965068	13.057398	0.453281
	H	3.969826	13.749692	-0.301856
	H	3.477618	12.228429	0.106024
	H	4.938489	12.799536	0.662484
$[B_{12}H_{11}NCCH_3NH_3*NH_3]^-$ **TS2**	N	4.090432	14.38008	2.64598
	C	3.441131	13.974575	1.645227
	C	1.947479	13.889263	1.570494
	H	1.570746	14.476952	0.725249
	H	1.508516	14.266258	2.494016
	H	1.629435	12.852448	1.415203
	B	6.725704	15.092852	1.540539
	H	6.346447	15.358146	0.432448
	B	5.584295	14.518851	2.798961
	B	6.825599	13.417003	2.124606
	H	6.523911	12.479494	1.432028
	B	8.311184	14.34734	1.838733
	H	9.057523	14.076621	0.937369
	B	8.004683	16.027249	2.338669
	H	8.537144	16.957261	1.794442
	B	6.330277	16.131601	2.93318
	H	5.667638	17.125863	2.804425
	B	6.199856	15.101349	4.374449
	H	5.432565	15.364143	5.260526
	B	6.490688	13.422839	3.873734
	H	5.940347	12.497338	4.407382
	B	8.165555	13.311206	3.279743
	H	8.809585	12.304374	3.402257
	B	8.902881	14.926902	3.411269
	H	10.076373	15.072369	3.626361
	B	7.682438	16.031002	4.088053
	H	7.987076	16.963236	4.781669
	B	7.782216	14.354585	4.670283
	H	8.15753	14.087748	5.780386
	N	4.073633	13.594234	0.376856
	H	4.085307	14.544477	-0.339636
	H	3.55911	12.847742	-0.090659
	H	5.036148	13.284268	0.525771
	N	4.073456	15.71026	-1.149906
	H	4.768369	15.635635	-1.889121
	H	4.299087	16.540567	-0.607401
	H	3.170375	15.865927	-1.591051
$[B_{12}H_{11}NCCH_3NH_2*NH_4]^-$ **Comp1**	N	4.069523	14.167713	2.486378
	C	3.447925	13.713125	1.470484
	C	1.95546	13.510001	1.477223
	H	1.483192	14.04453	0.645398
	H	1.534064	13.86636	2.41744
	H	1.716957	12.44698	1.358684
	B	6.774768	14.732906	1.349267
	H	6.492619	14.776381	0.183025
	B	5.549285	14.391525	2.630385

	B	6.836713	13.201266	2.248495
	H	6.565001	12.151034	1.727044
	B	8.338554	14.080111	1.870956
	H	9.13485	13.656917	1.077359
	B	8.003411	15.822347	2.015585
	H	8.563359	16.63142	1.325672
	B	6.298124	16.011993	2.487714
	H	5.644182	16.968123	2.149068
	B	6.093926	15.280161	4.090081
	H	5.280537	15.698351	4.869108
	B	6.420214	13.543681	3.943681
	H	5.84055	12.739201	4.623309
	B	8.120188	13.341299	3.472459
	H	8.765986	12.389301	3.819712
	B	8.844992	14.963754	3.327018
	H	10.004964	15.166898	3.568317
	B	7.585535	16.161863	3.71
	H	7.846935	17.216082	4.224374
	B	7.660876	14.630807	4.610231
	H	7.973061	14.592565	5.770537
	N	4.031742	13.433428	0.222101
	H	4.142254	15.25747	-0.300528
	H	3.500532	12.800394	-0.36338
	H	5.015047	13.189372	0.259467
	N	4.264608	16.293637	-0.303011
	H	4.823463	16.567151	-1.110861
	H	4.757823	16.575309	0.551328
	H	3.360961	16.763212	-0.345652
$[B_{12}H_{11}NHCCH_3NH_2{*}NH_3]^-$ **Comp2**	N	4.121121	14.440067	1.977155
	C	3.490593	13.62336	1.171839
	C	2.036438	13.823734	0.885084
	H	1.89583	13.991086	-0.186082
	H	1.643842	14.679411	1.433911
	H	1.476272	12.927883	1.165645
	B	6.904711	14.673159	1.168615
	H	6.680758	14.604001	-0.009104
	B	5.593369	14.522246	2.361111
	B	6.808866	13.231105	2.217954
	H	6.556815	12.139123	1.785353
	B	8.377242	13.999346	1.899853
	H	9.213641	13.453783	1.232901
	B	8.136304	15.761014	1.854384
	H	8.808644	16.470837	1.155735
	B	6.413729	16.084289	2.13861
	H	5.827511	17.011303	1.648092
	B	6.010569	15.512636	3.776458
	H	5.149454	16.042859	4.422963
	B	6.251568	13.749598	3.82599
	H	5.560077	13.042057	4.50713
	B	7.976396	13.429472	3.540392
	H	8.531349	12.48228	4.027578
	B	8.798146	14.991424	3.315998
	H	9.939248	15.158435	3.651356
	B	7.580557	16.280197	3.462637
	H	7.852155	17.362243	3.906972
	B	7.483255	14.839414	4.505009

	H	7.683367	14.903289	5.687768
	N	4.094034	12.600056	0.572293
	H	3.532886	15.208845	2.344481
	H	3.56425	11.985451	-0.02629
	H	5.06697	12.389632	0.753004
	N	2.681937	16.762428	3.010649
	H	1.814848	17.118708	2.619624
	H	3.395412	17.469448	2.853778
	H	2.553017	16.698926	4.016546
$[B_{12}H_{11}NHCCH_3NH_2]^-$ **Prod**	N	4.144857	14.033897	2.288244
	H	3.809372	14.433109	1.42069
	N	3.533721	12.628864	4.016461
	H	4.432557	12.72179	4.47023
	C	3.271168	13.245554	2.872111
	C	1.927743	13.023457	2.25849
	H	1.764191	11.954394	2.103136
	H	1.841679	13.542232	1.304307
	H	1.155203	13.393289	2.937451
	B	6.467075	15.490424	1.610148
	H	5.992495	15.818246	0.556706
	B	5.547054	14.460259	2.719264
	B	6.987431	13.811629	1.902137
	H	6.886156	12.968956	1.053096
	B	8.190746	15.115851	1.824358
	H	8.972706	15.194697	0.916037
	B	7.490404	16.563983	2.589634
	H	7.774488	17.671956	2.223801
	B	5.852931	16.158703	3.143258
	H	4.955859	16.95671	3.159191
	B	5.984659	14.89138	4.38598
	H	5.190651	14.806013	5.282768
	B	6.688688	13.438238	3.616647
	H	6.399972	12.330037	3.978229
	B	8.326638	13.850002	3.066722
	H	9.20209	13.027846	3.044288
	B	8.639687	15.547276	3.491719
	H	9.742758	15.931871	3.769569
	B	7.194691	16.19108	4.304191
	H	7.26467	17.033073	5.157993
	B	7.707165	14.512094	4.599514
	H	8.144664	14.161717	5.661362
	H	2.839264	12.028885	4.434577

Tetrahydrofuran Solution:

Compound	Atom	x	y	z
$[B_{12}H_{11}NCCH_3]^-$ **Init**	N	4.282256	13.826371	2.009571
	C	3.197042	13.58918	1.725937
	C	1.829079	13.291858	1.367813
	H	1.81295	12.847319	0.370681
	H	1.250663	14.218212	1.37299
	H	1.416548	12.590618	2.096383
	B	6.839053	14.715084	1.158854
	H	6.494481	14.873757	0.0203
	B	5.708898	14.134723	2.382833
	B	7.011233	13.071784	1.852753

	H	6.788596	12.091723	1.197569
	B	8.440591	14.103166	1.626194
	H	9.274796	13.840621	0.803711
	B	7.98016	15.785716	1.999686
	H	8.485719	16.720922	1.441377
	B	6.263713	15.805968	2.45889
	H	5.524351	16.719084	2.21675
	B	6.080108	14.838533	3.955645
	H	5.213871	15.083628	4.748803
	B	6.543092	13.148233	3.581091
	H	5.998352	12.221982	4.115241
	B	8.257666	13.140353	3.115873
	H	8.961307	12.196589	3.350644
	B	8.858053	14.814031	3.204731
	H	9.995059	15.059505	3.503951
	B	7.513241	15.861855	3.71909
	H	7.690883	16.849919	4.377451
	B	7.685197	14.227075	4.40978
	H	7.984882	14.052786	5.55951
**NH_3**	N	2.053074	23.982792	5.575194
	H	2.053144	24.925637	5.951753
	H	2.869856	23.511498	5.951738
	H	1.236346	23.511449	5.951723
$[B_{12}H_{11}NCCH_3*NH_3]^-$ **TS1**	N	4.104378	13.988191	2.46322
	C	3.212862	13.639504	1.782925
	C	1.770038	13.453973	1.598152
	H	1.430894	14.040013	0.741555
	H	1.260365	13.78855	2.504182
	H	1.552707	12.399711	1.415835
	B	6.65123	14.75803	1.456031
	H	6.248388	14.932291	0.336428
	B	5.557078	14.234585	фев.09
	B	6.798126	13.121291	2.148414
	H	6.502127	12.142347	1.515646
	B	8.254564	14.084938	1.819986
	H	9.021206	13.782087	0.946117
	B	7.887524	15.78546	2.207005
	H	8.393439	16.696546	1.608954
	B	6.205375	15.877649	2.779256
	H	5.499092	16.8274	2.575847
	B	6.083589	14.933118	4.289579
	H	5.288525	15.223483	5.140918
	B	6.443323	13.228162	3.89931
	H	5.904423	12.323941	4.477583
	B	8.124823	13.139902	3.325361
	H	8.800169	12.165816	3.521853
	B	8.803476	14.785986	3.360683
	H	9.967927	14.984088	3.58237
	B	7.540827	15.891869	3.952715
	H	7.804777	16.87622	4.588299
	B	7.687507	14.257474	4.643948
	H	8.057587	14.074898	5.772158
	N	3.907039	12.963761	-0.029771
	H	4.36623	13.754624	-0.475236

	H	3.257433	12.552587	-0.695201
	H	4.62718	12.277628	0.184919
$[B_{12}H_{11}NCCH_3NH_3]^-$ **Int**	N	3.948049	14.110267	2.628672
	C	3.291063	13.629303	1.69038
	C	1.803963	13.520375	1.569051
	H	1.451465	13.997018	0.647571
	H	1.339803	14.00833	2.426177
	H	1.492496	12.470311	1.542994
	B	6.53383	14.798314	1.469741
	H	6.109691	15.00679	0.361971
	B	5.44193	14.26743	2.78208
	B	6.674079	13.151438	2.123226
	H	6.373593	12.174766	1.479805
	B	8.137155	14.086003	1.759558
	H	8.862967	13.78412	0.850854
	B	7.817323	15.78215	2.196022
	H	8.32206	16.695319	1.599284
	B	6.156578	15.892264	2.82871
	H	5.472549	16.867919	2.671404
	B	6.071328	14.920884	4.316208
	H	5.316582	15.209877	5.204812
	B	6.372678	13.223321	3.878649
	H	5.841797	12.313089	4.456766
	B	8.036618	13.108918	3.248077
	H	8.694182	12.113912	3.395845
	B	8.753904	14.738278	3.293584
	H	9.930128	14.905647	3.476536
	B	7.536145	15.855059	3.95316
	H	7.843801	16.819992	4.599415
	B	7.669625	14.20515	4.602717
	H	8.074316	13.987635	5.713107
	N	3.982147	13.064933	0.45735
	H	4.012168	13.75878	-0.294903
	H	3.502162	12.238141	0.096221
	H	4.95229	12.802277	0.688271
$[B_{12}H_{11}NCCH_3NH_3{*}NH_3]^-$ **TS2**	N	4.083629	14.351041	2.640146
	C	3.432547	13.948004	1.646824
	C	1.935203	13.836534	1.590607
	H	1.532479	14.424126	0.756698
	H	1.508199	14.201804	2.525091
	H	1.627096	12.795907	1.43735
	B	6.719442	15.064508	1.519561
	H	июн.42	15.313583	0.401668
	B	5.57667	14.508703	2.786538
	B	6.818611	13.398556	2.128371
	H	июн.63	12.448961	1.448358
	B	8.304363	14.326442	1.83041
	H	9.050963	14.042953	0.931402
	B	7.993457	16.014175	2.303098
	H	8.523258	16.936997	1.741573
	B	6.316419	16.125137	2.893097
	H	5.650113	17.116207	2.743885
	B	6.186701	15.117731	4.351793

	H	5.415096	15.392723	5.231368
	B	6.48188	13.431355	мар.78
	H	5.929796	12.512677	4.423748
	B	8.157498	13.312978	3.287093
	H	8.802719	12.307805	3.425833
	B	8.892086	14.932563	3.394281
	H	10.065949	15.083788	3.608071
	B	7.668084	16.045665	4.051709
	H	7.969739	16.990772	4.730575
	B	7.770634	14.378283	4.660682
	H	8.144738	14.128689	5.776008
	N	4.058082	13.604795	0.365022
	H	4.094143	14.616987	-0.34987
	H	3.546542	12.871634	-0.124756
	H	5.01973	13.287536	0.508659
	N	4.142868	15.757896	-1.074685
	H	4.868188	15.691406	-1.785622
	H	4.374188	16.549342	-0.477528
	H	3.263128	15.963794	-1.542323
$[B_{12}H_{11}NCCH_3NH_2*NH_4]^-$ **Comp1**	N	4.065555	14.156403	2.478202
	C	3.435946	13.709326	1.472161
	C	1.943328	13.486972	1.492503
	H	1.453346	14.014588	0.665574
	H	1.532348	13.841264	2.43865
	H	1.712614	12.421027	1.381673
	B	6.770251	14.729285	1.338719
	H	6.496581	14.766584	0.168271
	B	5.544999	14.39074	2.62133
	B	6.83232	13.201725	2.24212
	H	6.561482	12.148336	1.723259
	B	8.333922	14.082826	1.863833
	H	9.130346	13.659336	1.069081
	B	7.994378	15.825167	2.002107
	H	8.549851	16.634926	1.307141
	B	6.288637	16.010785	2.473898
	H	5.63356	16.970517	2.136421
	B	6.081975	15.285354	4.078593
	H	5.263573	15.704486	4.852475
	B	6.412696	13.548338	3.93715
	H	5.830998	12.74433	4.61645
	B	8.113839	13.348543	3.467605
	H	8.760758	12.397536	3.817608
	B	8.835747	14.972602	3.317834
	H	9.995649	15.179734	3.559172
	B	7.573199	16.169357	3.695765
	H	7.829912	17.228031	4.205008
	B	7.650688	14.641139	4.600944
	H	7.960989	14.607796	5.762434
	N	4.013047	13.463857	0.20338
	H	4.177918	15.247245	-0.278562
	H	3.48357	12.831882	-0.38614
	H	4.991741	13.195837	0.241588
	N	4.328037	16.285453	-0.260428
	H	4.907949	16.562589	-1.052018
	H	4.81414	16.535735	0.611793

	H	3.436291	16.777016	-0.307126
$[B_{12}H_{11}NHCCH_3NH_2*NH_3]^-$ **Comp2**	N	4.117205	14.433958	1.972136
	C	3.490674	13.612039	1.173145
	C	2.029826	13.797387	0.893151
	H	1.876412	13.956358	-0.177816
	H	1.636522	14.655024	1.439492
	H	1.476114	12.901253	1.186746
	B	6.898437	14.676805	1.164855
	H	6.670416	14.607066	-0.013746
	B	5.591273	14.522257	2.358791
	B	6.806814	13.233462	2.213567
	H	6.559298	12.13828	1.781349
	B	8.372618	14.006244	1.895693
	H	9.210153	13.462736	1.227069
	B	8.126283	15.767817	1.85182
	H	8.795925	16.481016	1.152686
	B	6.402808	16.086333	2.13672
	H	5.812841	17.011852	1.643881
	B	6.001668	15.510881	3.774849
	H	5.138847	16.035285	4.426346
	B	6.246442	13.747943	3.822249
	H	5.553213	13.037325	4.500752
	B	7.972737	13.43306	3.536216
	H	8.52931	12.485195	4.022192
	B	8.789894	14.998053	3.313174
	H	9.931181	15.167913	3.648845
	B	7.568942	16.283665	3.461396
	H	7.836067	17.367322	3.906888
	B	7.475419	14.840851	4.502546
	H	7.673815	14.904146	5.686397
	N	4.104477	12.595892	0.569808
	H	3.524837	15.198129	2.336197
	H	3.585004	11.970026	-0.025445
	H	5.08138	12.397505	0.74863
	N	2.699859	16.78468	3.01286
	H	1.951609	17.293902	2.551752
	H	3.526786	17.376603	2.98999
	H	2.440078	16.684887	3.990214
$[B_{12}H_{11}NHCCH_3NH_2]^-$ **Prod**	N	4.147089	14.035072	2.28479
	H	3.818573	14.431314	1.413254
	N	3.539137	12.644026	4.020229
	H	4.439592	12.742543	4.471369
	C	3.274433	13.251461	2.869995
	C	1.92785	13.019197	2.258246
	H	1.77084	11.949269	2.099285
	H	1.836406	13.537623	1.303843
	H	1.150411	13.38251	2.935492
	B	6.465922	15.494692	1.611807
	H	5.986673	15.826804	0.559902
	B	5.551597	14.460612	2.718458
	B	6.985406	13.81317	1.894327
	H	6.87843	12.973473	1.040994
	B	8.189238	15.117737	1.821239

	H	8.969692	15.200336	0.910954
	B	7.490254	16.562955	2.595269
	H	7.773258	17.673658	2.23427
	B	5.853091	16.155541	3.14948
	H	4.952549	16.951773	3.170045
	B	5.98717	14.881419	4.386637
	H	5.192117	14.794338	5.284712
	B	6.689806	13.431359	3.608623
	H	6.40134	12.318698	3.962817
	B	8.326477	13.845113	3.057574
	H	9.201101	13.021023	3.029687
	B	8.64028	15.540655	3.490566
	H	9.744502	15.923582	3.769129
	B	7.196654	16.181354	4.308965
	H	7.266682	17.019304	5.167853
	B	7.709809	14.500164	4.595304
	H	8.147911	14.143331	5.655863
	H	2.851079	12.044302	4.448127
