# Supplementary material for: Primary Amine Nucleophilic Addition to Nitrilium Closo-Dodecaborate [B12H11NCCH3]−: A Simple and Effective Route to the New BNCT Drug Design
Source: Int J Mol Sci. 2021 Dec 13;22(24):13391. doi: 10.3390/ijms222413391 (PMC8707455; doi:10.3390/ijms222413391)

checkCIF/PLATON report

Structure factors have been supplied for datablock(s) Zh202

THIS REPORT IS FOR GUIDANCE ONLY. IF USED AS PART OF A REVIEW PROCEDURE FOR PUBLICATION, IT SHOULD NOT REPLACE THE EXPERTISE OF AN EXPERIENCED CRYSTALLOGRAPHIC REFEREE.

No syntax errors found. CIF dictionary Interpreting this report

Datablock: Zh202

Bond precision: C-C = 0.0035 A Wavelength=0.71073

Cell:	a=11.1175(5)	b=12.6408(5)	c=20.8691(10)
	alpha=90	beta=92.663(2)	gamma=90
Temperature:	296 K		

	Calculated	Reported
Volume	2929.7(2)	2929.7(2)
Space group	P 21/n	P 21/n
Hall group	-P 2yn	-P 2yn
Moiety formula	C16 H36 N, C2 H17 B12 N2	?
Sum formula	C18 H53 B12 N3	C18 H53 B12 N3
Mr	441.35	441.35
Dx,g cm-3	1.001	1.001
Z	4	4
Mu (mm-1)	0.052	0.052
F000	968.0	968.0
F000’	968.17	
h,k,lmax	14,16,27	14,16,27
Nref	6737	6697
Tmin,Tmax	0.986,0.990	
Tmin’	0.981	

Correction method= Not given

Data completeness= 0.994 Theta(max)= 27.499

R(reflections)= 0.0619(4159) wR2(reflections)= 0.1897(6697)

S = 1.015 Npar= 332

The following ALERTS were generated. Each ALERT has the format
test-name_ALERT_alert-type_alert-level.
Click on the hyperlinks for more details of the test.

Alert level B

```
PLAT910_ALERT_3_B Missing # of FCF Reflection(s) Below Theta(Min).        24 Note
```

Author Response: Closed by beamstop.

Alert level C

```
PLAT220_ALERT_2_C NonSolvent Resd 1  C   Ueq(max) / Ueq(min) Range        3.1 Ratio
PLAT230_ALERT_2_C Hirshfeld Test Diff for    C42      --C43      .        6.0 s.u.
PLAT230_ALERT_2_C Hirshfeld Test Diff for    C43      --C44      .        5.2 s.u.
PLAT242_ALERT_2_C Low    MainMol Ueq as Compared to Neighbors of        C33 Check
PLAT242_ALERT_2_C Low    MainMol Ueq as Compared to Neighbors of         C1 Check
PLAT414_ALERT_2_C Short Intra D-H..H-X       H2       ..H2A              1.94 Ang.
                                                  x,y,z  =      1_555 Check
PLAT905_ALERT_3_C Negative K value in the Analysis of Variance ...     -0.425 Report
PLAT911_ALERT_3_C Missing FCF Refl Between Thmin & STh/L=    0.600          8 Report
```

Alert level G

```
PLAT007_ALERT_5_G Number of Unrefined Donor-H Atoms ..............          3 Report
PLAT883_ALERT_1_G No Info/Value for _atom_sites_solution_primary .     Please Do !
PLAT912_ALERT_4_G Missing # of FCF Reflections Above STh/L=  0.600          9 Note
PLAT933_ALERT_2_G Number of OMIT Records in Embedded .res File ...          8 Note
PLAT978_ALERT_2_G Number C-C Bonds with Positive Residual Density.          1 Info
```

---

0 **ALERT level A** = Most likely a serious problem - resolve or explain
1 **ALERT level B** = A potentially serious problem, consider carefully
8 **ALERT level C** = Check. Ensure it is not caused by an omission or oversight
5 **ALERT level G** = General information/check it is not something unexpected

1 ALERT type 1 CIF construction/syntax error, inconsistent or missing data
8 ALERT type 2 Indicator that the structure model may be wrong or deficient
3 ALERT type 3 Indicator that the structure quality may be low
1 ALERT type 4 Improvement, methodology, query or suggestion
1 ALERT type 5 Informative message, check

It is advisable to attempt to resolve as many as possible of the alerts in all categories. Often the minor alerts point to easily fixed oversights, errors and omissions in your CIF or refinement strategy, so attention to these fine details can be worthwhile. In order to resolve some of the more serious problems it may be necessary to carry out additional measurements or structure refinements. However, the purpose of your study may justify the reported deviations and the more serious of these should normally be commented upon in the discussion or experimental section of a paper or in the "special_details" fields of the CIF. checkCIF was carefully designed to identify outliers and unusual parameters, but every test has its limitations and alerts that are not important in a particular case may appear. Conversely, the absence of alerts does not guarantee there are no aspects of the results needing attention. It is up to the individual to critically assess their own results and, if necessary, seek expert advice.

Publication of your CIF in IUCr journals

A basic structural check has been run on your CIF. These basic checks will be run on all CIFs submitted for publication in IUCr journals (*Acta Crystallographica*, *Journal of Applied Crystallography*, *Journal of Synchrotron Radiation*); however, if you intend to submit to *Acta Crystallographica Section C* or *E* or *IUCrData*, you should make sure that full publication checks are run on the final version of your CIF prior to submission.

Publication of your CIF in other journals

Please refer to the *Notes for Authors* of the relevant journal for any special instructions relating to CIF submission.

PLATON version of 22/12/2019; check.def file version of 13/12/2019

Datablock Zh202 - ellipsoid plot

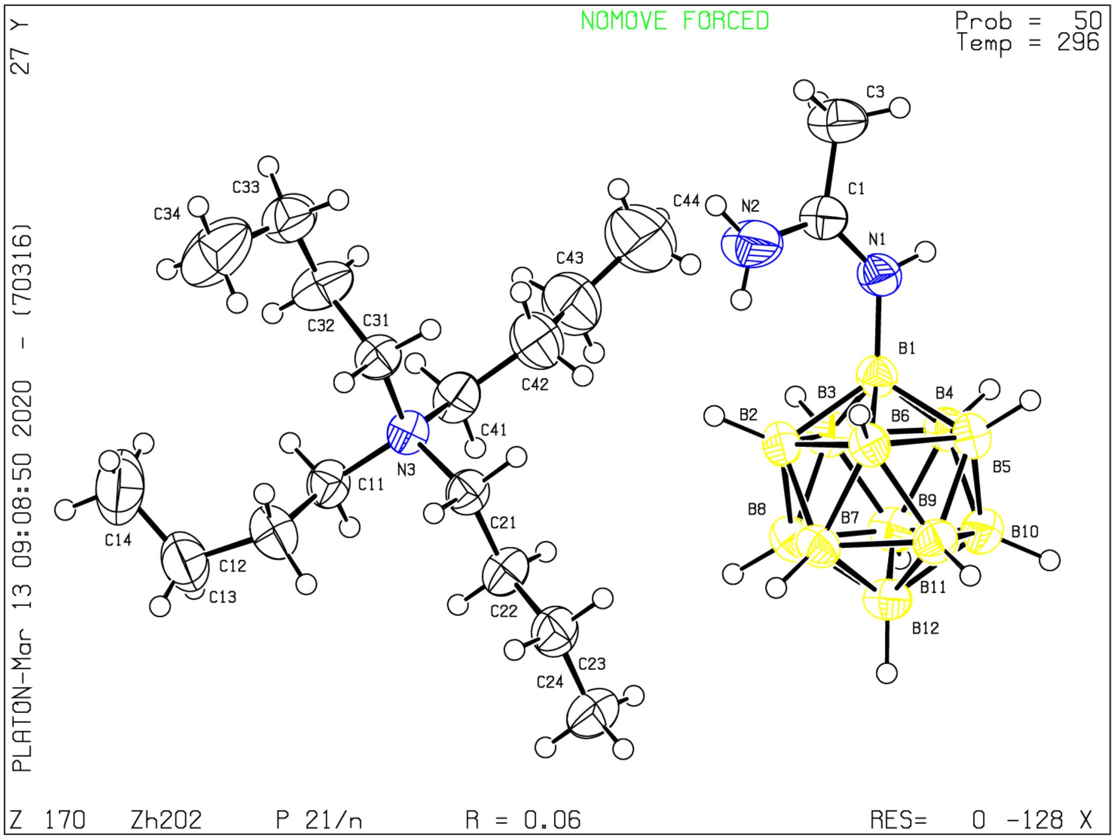

Supplement: Supplementary file 1 [file checkcif_CCDC 2122676.pdf › checkcif_CCDC 2122676.pdf]
