# Supplementary material for: Primary Amine Nucleophilic Addition to Nitrilium Closo-Dodecaborate [B12H11NCCH3]−: A Simple and Effective Route to the New BNCT Drug Design
Source: Int J Mol Sci. 2021 Dec 13;22(24):13391. doi: 10.3390/ijms222413391 (PMC8707455; doi:10.3390/ijms222413391)

checkCIF/PLATON report

Structure factors have been supplied for datablock(s) Zh361

THIS REPORT IS FOR GUIDANCE ONLY. IF USED AS PART OF A REVIEW PROCEDURE FOR PUBLICATION, IT SHOULD NOT REPLACE THE EXPERTISE OF AN EXPERIENCED CRYSTALLOGRAPHIC REFEREE.

No syntax errors found. CIF dictionary Interpreting this report

Datablock: Zh361

```
Bond precision:  C-C = 0.0091 A          Wavelength=0.71073

Cell:           a=11.4609(12)      b=18.020(2)       c=19.186(3)
                alpha=112.896(7)   beta=100.698(8)   gamma=101.699(5)
Temperature:    296 K

                  Calculated                Reported
Volume            3417.3(8)                 3417.2(8)
Space group       P -1                      P -1
Hall group        -P 1                      -P 1
Moiety formula    C8 H21 B12 N2, C16 H36 N  ?
Sum formula       C24 H57 B12 N3            C24 H57 B12 N3
Mr                517.45                    517.44
Dx,g cm-3         1.006                     1.006
Z                 4                         4
Mu (mm-1)         0.053                     0.053
F000              1128.0                    1128.0
F000'             1128.23
h,k,lmax          14,23,24                  14,23,24
Nref              15690                     15532
Tmin,Tmax         0.981,0.998
Tmin'             0.979

Correction method= Not given

Data completeness= 0.990          Theta(max)= 27.498

R(reflections)= 0.0891( 4265)     wR2(reflections)= 0.2784( 15532)

S = 0.903                  Npar= 769
```

The following ALERTS were generated. Each ALERT has the format
test-name_ALERT_alert-type_alert-level.
Click on the hyperlinks for more details of the test.

Alert level A

```
PLAT026_ALERT_3_A Ratio Observed / Unique Reflections (too) Low ..        27% Check
```

Author Response: Small crystal.

```
PLAT910_ALERT_3_A Missing # of FCF Reflection(s) Below Theta(Min).         57 Note
```

Author Response: Closed by beamstop.

Alert level B

```
PLAT234_ALERT_4_B Large Hirshfeld Difference C12      --C13      .       0.27 Ang.
PLAT234_ALERT_4_B Large Hirshfeld Difference C13      --C14      .       0.28 Ang.
```

Alert level C

```
PLAT084_ALERT_3_C High wR2 Value (i.e. > 0.25) ...................       0.28 Report
PLAT202_ALERT_3_C Isotropic non-H Atoms in Anion/Solvent .........          1 Check
              C54A
PLAT221_ALERT_2_C Solv./Anion  Resd 3  C   Ueq(max)/Ueq(min) Range        4.4 Ratio
PLAT223_ALERT_4_C Solv./Anion  Resd 3  H   Ueq(max)/Ueq(min) Range        5.5 Ratio
PLAT223_ALERT_4_C Solv./Anion  Resd 4  H   Ueq(max)/Ueq(min) Range        4.6 Ratio
PLAT230_ALERT_2_C Hirshfeld Test Diff for    C33      --C34      .        6.6 s.u.
PLAT230_ALERT_2_C Hirshfeld Test Diff for    C63      --C64      .        5.6 s.u.
PLAT234_ALERT_4_C Large Hirshfeld Difference C32      --C33      .       0.17 Ang.
PLAT234_ALERT_4_C Large Hirshfeld Difference C41      --C42      .       0.16 Ang.
PLAT241_ALERT_2_C High   ’MainMol’ Ueq as Compared to Neighbors of        C83 Check
PLAT242_ALERT_2_C Low    ’MainMol’ Ueq as Compared to Neighbors of        C3A Check
PLAT242_ALERT_2_C Low    ’MainMol’ Ueq as Compared to Neighbors of        C11 Check
PLAT242_ALERT_2_C Low    ’MainMol’ Ueq as Compared to Neighbors of        C12 Check
PLAT242_ALERT_2_C Low    ’MainMol’ Ueq as Compared to Neighbors of        C21 Check
PLAT242_ALERT_2_C Low    ’MainMol’ Ueq as Compared to Neighbors of        C31 Check
PLAT242_ALERT_2_C Low    ’MainMol’ Ueq as Compared to Neighbors of        C52 Check
PLAT242_ALERT_2_C Low    ’MainMol’ Ueq as Compared to Neighbors of        C61 Check
PLAT242_ALERT_2_C Low    ’MainMol’ Ueq as Compared to Neighbors of        C63 Check
PLAT242_ALERT_2_C Low    ’MainMol’ Ueq as Compared to Neighbors of        C71 Check
PLAT242_ALERT_2_C Low    ’MainMol’ Ueq as Compared to Neighbors of        C73 Check
PLAT242_ALERT_2_C Low    ’MainMol’ Ueq as Compared to Neighbors of        C82 Check
PLAT260_ALERT_2_C Large Average Ueq of Residue Including       N3      0.145 Check
PLAT260_ALERT_2_C Large Average Ueq of Residue Including       N4      0.108 Check
PLAT331_ALERT_2_C Small Aver Phenyl C-C Dist C3       --C8       .       1.37 Ang.
PLAT331_ALERT_2_C Small Aver Phenyl C-C Dist C3A      --C8A      .       1.37 Ang.
PLAT340_ALERT_3_C Low Bond Precision on  C-C Bonds ...............    0.00906 Ang.
PLAT360_ALERT_2_C Short  C(sp3)-C(sp3) Bond  C13      - C14      .       1.42 Ang.
PLAT420_ALERT_2_C D-H Without Acceptor       N1       --H1B      .     Please Check
PLAT906_ALERT_3_C Large K Value in the Analysis of Variance ......     14.314 Check
PLAT906_ALERT_3_C Large K Value in the Analysis of Variance ......      2.401 Check
PLAT911_ALERT_3_C Missing FCF Refl Between Thmin & STh/L=    0.600         27 Report
```

Alert level G

```
PLAT002_ALERT_2_G Number of Distance or Angle Restraints on AtSite         22 Note
PLAT007_ALERT_5_G Number of Unrefined Donor-H Atoms ..............          4 Report
PLAT168_ALERT_4_G The CIF-Embedded .res File Contains EXYZ Records          3 Report
PLAT171_ALERT_4_G The CIF-Embedded .res File Contains EADP Records          3 Report
PLAT176_ALERT_4_G The CIF-Embedded .res File Contains SADI Records          1 Report
PLAT300_ALERT_4_G Atom Site Occupancy of C23A       Constrained at        0.5 Check
```

```
PLAT300_ALERT_4_G Atom Site Occupancy of C23B       Constrained at       0.5 Check
PLAT300_ALERT_4_G Atom Site Occupancy of C24A       Constrained at       0.5 Check
PLAT300_ALERT_4_G Atom Site Occupancy of C24B       Constrained at       0.5 Check
PLAT300_ALERT_4_G Atom Site Occupancy of C43A       Constrained at       0.5 Check
PLAT300_ALERT_4_G Atom Site Occupancy of C43B       Constrained at       0.5 Check
PLAT300_ALERT_4_G Atom Site Occupancy of C44A       Constrained at       0.5 Check
PLAT300_ALERT_4_G Atom Site Occupancy of C44B       Constrained at       0.5 Check
PLAT300_ALERT_4_G Atom Site Occupancy of H23A       Constrained at       0.5 Check
PLAT300_ALERT_4_G Atom Site Occupancy of H23B       Constrained at       0.5 Check
PLAT300_ALERT_4_G Atom Site Occupancy of H23C       Constrained at       0.5 Check
PLAT300_ALERT_4_G Atom Site Occupancy of H23D       Constrained at       0.5 Check
PLAT300_ALERT_4_G Atom Site Occupancy of H24A       Constrained at       0.5 Check
PLAT300_ALERT_4_G Atom Site Occupancy of H24B       Constrained at       0.5 Check
PLAT300_ALERT_4_G Atom Site Occupancy of H24C       Constrained at       0.5 Check
PLAT300_ALERT_4_G Atom Site Occupancy of H24D       Constrained at       0.5 Check
PLAT300_ALERT_4_G Atom Site Occupancy of H24E       Constrained at       0.5 Check
PLAT300_ALERT_4_G Atom Site Occupancy of H24F       Constrained at       0.5 Check
PLAT300_ALERT_4_G Atom Site Occupancy of H43A       Constrained at       0.5 Check
PLAT300_ALERT_4_G Atom Site Occupancy of H43B       Constrained at       0.5 Check
PLAT300_ALERT_4_G Atom Site Occupancy of H43C       Constrained at       0.5 Check
PLAT300_ALERT_4_G Atom Site Occupancy of H43D       Constrained at       0.5 Check
PLAT300_ALERT_4_G Atom Site Occupancy of H44A       Constrained at       0.5 Check
PLAT300_ALERT_4_G Atom Site Occupancy of H44B       Constrained at       0.5 Check
PLAT300_ALERT_4_G Atom Site Occupancy of H44C       Constrained at       0.5 Check
PLAT300_ALERT_4_G Atom Site Occupancy of H44D       Constrained at       0.5 Check
PLAT300_ALERT_4_G Atom Site Occupancy of H44E       Constrained at       0.5 Check
PLAT300_ALERT_4_G Atom Site Occupancy of H44F       Constrained at       0.5 Check
PLAT302_ALERT_4_G Anion/Solvent/Minor-Residue Disorder (Resd  3  )       24% Note
PLAT302_ALERT_4_G Anion/Solvent/Minor-Residue Disorder (Resd  4  )       12% Note
PLAT412_ALERT_2_G Short Intra XH3 .. XHn     H21A     ..H24F     .      2.13 Ang.
                                                        x,y,z  =     1_555 Check
PLAT412_ALERT_2_G Short Intra XH3 .. XHn     H41A     ..H44E     .      2.14 Ang.
                                                        x,y,z  =     1_555 Check
PLAT412_ALERT_2_G Short Intra XH3 .. XHn     H51A     ..H54F     .      2.00 Ang.
                                                        x,y,z  =     1_555 Check
PLAT414_ALERT_2_G Short Intra D-H..H-X       H2C      ..H5A             2.10 Ang.
                                                        x,y,z  =     1_555 Check
PLAT415_ALERT_2_G Short Inter D-H..H-X       H1C      ..H4       .      2.07 Ang.
                                                        x,y,z  =     1_555 Check
PLAT720_ALERT_4_G Number of Unusual/Non-Standard Labels ..........         8 Note
PLAT860_ALERT_3_G Number of Least-Squares Restraints .............        55 Note
PLAT883_ALERT_1_G No Info/Value for _atom_sites_solution_primary .     Please Do !
PLAT912_ALERT_4_G Missing # of FCF Reflections Above STh/L=  0.600        76 Note
PLAT933_ALERT_2_G Number of OMIT Records in Embedded .res File ...         3 Note
PLAT941_ALERT_3_G Average HKL Measurement Multiplicity ...........       2.0 Low
PLAT978_ALERT_2_G Number C-C Bonds with Positive Residual Density.         1 Info
```

---

   2 **ALERT level A** = Most likely a serious problem - resolve or explain
   2 **ALERT level B** = A potentially serious problem, consider carefully
  31 **ALERT level C** = Check. Ensure it is not caused by an omission or oversight
  47 **ALERT level G** = General information/check it is not something unexpected

   1 ALERT type 1 CIF construction/syntax error, inconsistent or missing data
  29 ALERT type 2 Indicator that the structure model may be wrong or deficient
  10 ALERT type 3 Indicator that the structure quality may be low
  41 ALERT type 4 Improvement, methodology, query or suggestion
   1 ALERT type 5 Informative message, check

It is advisable to attempt to resolve as many as possible of the alerts in all categories. Often the minor alerts point to easily fixed oversights, errors and omissions in your CIF or refinement strategy, so attention to these fine details can be worthwhile. In order to resolve some of the more serious problems it may be necessary to carry out additional measurements or structure refinements. However, the purpose of your study may justify the reported deviations and the more serious of these should normally be commented upon in the discussion or experimental section of a paper or in the "special_details" fields of the CIF. checkCIF was carefully designed to identify outliers and unusual parameters, but every test has its limitations and alerts that are not important in a particular case may appear. Conversely, the absence of alerts does not guarantee there are no aspects of the results needing attention. It is up to the individual to critically assess their own results and, if necessary, seek expert advice.

Publication of your CIF in IUCr journals

A basic structural check has been run on your CIF. These basic checks will be run on all CIFs submitted for publication in IUCr journals (*Acta Crystallographica*, *Journal of Applied Crystallography*, *Journal of Synchrotron Radiation*); however, if you intend to submit to *Acta Crystallographica Section C* or *E* or *IUCrData*, you should make sure that full publication checks are run on the final version of your CIF prior to submission.

Publication of your CIF in other journals

Please refer to the *Notes for Authors* of the relevant journal for any special instructions relating to CIF submission.

PLATON version of 05/12/2020; check.def file version of 05/12/2020

Datablock Zh361 - ellipsoid plot

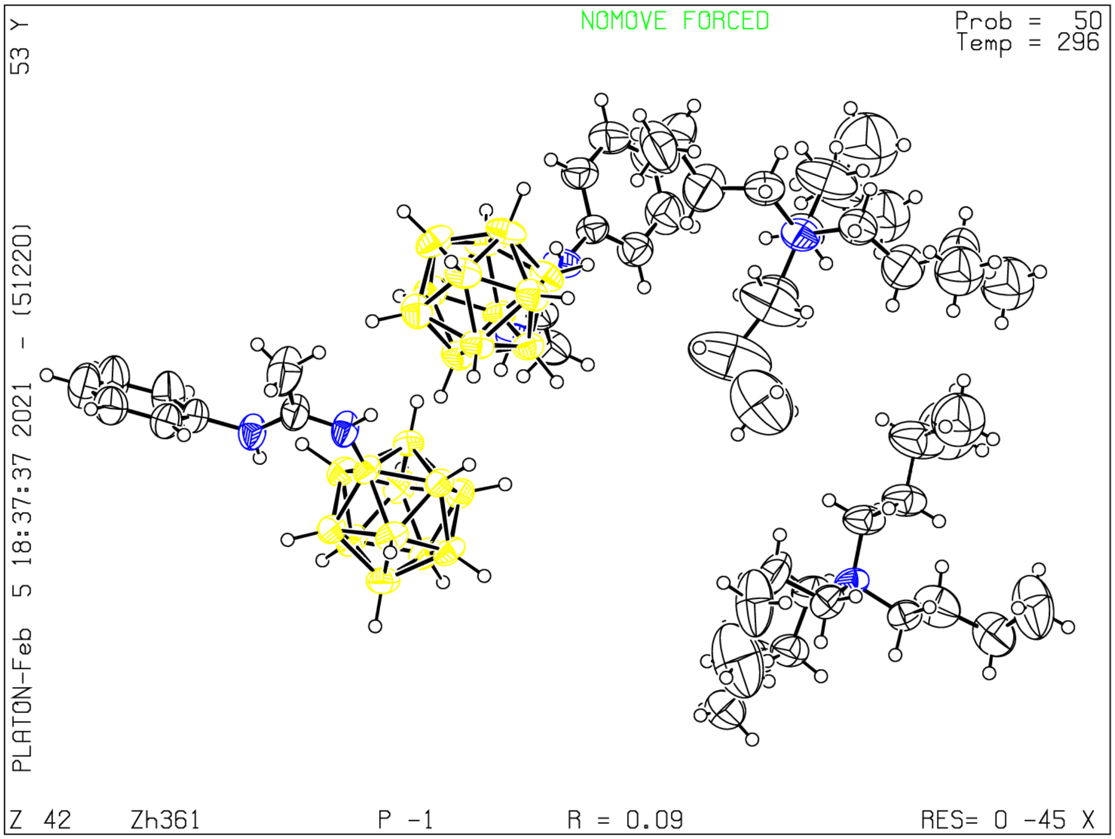

Supplement: Supplementary file 1 [file checkcif_CCDC 2122679.pdf › checkcif_CCDC 2122679.pdf]
